# *In Operando* Study of Charge Modulation in MoS_2_ Transistors by Excitonic Reflection Microscopy

**DOI:** 10.1021/acsnano.3c09337

**Published:** 2024-03-28

**Authors:** Nathan Ullberg, Arianna Filoramo, Stéphane Campidelli, Vincent Derycke

**Affiliations:** Université Paris-Saclay, CEA, CNRS, NIMBE, LICSEN, 91191 Gif-sur-Yvette, France

**Keywords:** TMD, MoS2, exciton, charge density, field-effect transistor, interference reflection microscopy, *in operando*

## Abstract

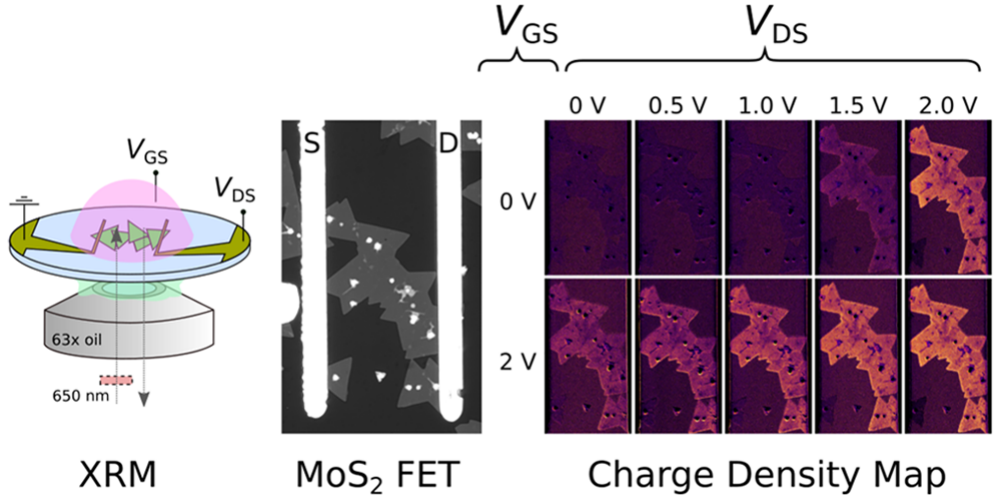

Monolayers of transition metal dichalcogenides (2D TMDs) experience strong modulation of their optical properties when the charge density is varied. Indeed, the transition from carriers composed mostly of excitons at low electron density to a situation in which trions dominate at high density is accompanied by a significant evolution of both the refractive index and the extinction coefficient. Using optical interference reflection microscopy at the excitonic wavelength, this (*n*, κ)–*q* relationship can be exploited to directly image the electron density in operating TMD devices. In this work, we show how this technique, which we call XRM (excitonic reflection microscopy), can be used to study charge distribution in MoS_2_ field-effect transistors with subsecond throughput, in wide-field mode. Complete maps of the charge distribution in the transistor channel at any drain and gate bias polarization point (*V*_DS_, *V*_GS_) are obtained, at ∼3 orders of magnitude faster than with scanning probe techniques such as KPFM. We notably show how the advantages of XRM enable real-time mapping of bias-dependent charge inhomogeneities, the study of resistive delays in 2D polycrystalline networks, and the evaluation of the *V*_DS_ vs *V*_GS_ competition to control the charge distribution in active devices.

## Introduction

Local control of the electron density in semiconductors is at the heart of modern electronics. The elementary building block of microprocessors are field-effect transistors (FETs) that rely on modulation of the charge density profile (CDP) in a semiconducting channel upon application of source-drain (*V*_DS_) and source-gate voltages (*V*_GS_). Beyond this cornerstone of information technology, charge modulation by a capacitive effect also plays a central role in sensors and optoelectronics. As a result, the capability to directly map the local variations of the charge density in operating devices is highly desirable. However, few techniques allow such *in operando* charge mapping. Kelvin probe force microscopy (KPFM) is typically the method of choice, as it can be used to image work function modulation in transistors and its evolution as a function of *V*_DS_ and *V*_GS_. Other techniques can be considered, in particular scanning gate microscopy,^1^ microwave impedance microscopy (MIM),^2^ and in some cases optical microspectroscopy methods like μ-Raman and μ-photoluminescence, thanks to the indirect link between the probed optical signal and the local charge density.^3^ Yet, all these techniques suffer from an extremely low throughput, typically several minutes per image at each (*V*_DS_, *V*_GS_) polarization point, which originates from either the scanning nature of the technique or the low signal of spectroscopic techniques that implies a long acquisition time at each pixel of the map. Developing techniques that are able to map charge density in wide-field without needing to scan are thus highly desirable and would allow device analysis in real time.

In that respect, two-dimensional transition metal dichalcogenides (2D TMDs) are of particular interest. These emerging nanomaterials are potential candidates for beyond-CMOS electronics,^4^ for applications in optoelectronics,^5^ photonics,^6^ and sensing,^7^ and most importantly, their optical properties are extremely sensitive to the charge density. Indeed, Mak et al.^8^ showed that in MoS_2_ monolayers, the optical absorption spectrum is dominated by exciton contributions at low charge density and by trions (charged excitons) upon electron accumulation. Later, several groups have shown that in most TMDs, the refractive index and extinction coefficient (*n*, κ) are affected by the charge density in extremely high proportions near exciton energies.^9,10^ Based on this relation between charge and complex refractive index, Scuri et al.^11^ imaged the charging of a MoSe_2_ flake on a SiO_2_/h-BN substrate. Zhu et al.^12^ imaged the voltage-dependent optical transmittance in MoS_2_ monolayers lying on an ITO substrate and proposed a calibration method to convert the optical signal into a charge density map. They demonstrated the use of this charge imaging technique as a powerful means to study protein binding on MoS_2_. Such biosensing capability was further confirmed in a different article by Zhu et al.,^13^ using a total internal reflectance configuration where the binding kinetics of protein molecules on MoS_2_ were measured. Zhao et al. also imaged charge in MoS_2_ using surface plasmon resonance imaging.^14^

In this context, we developed the use of an interference reflection microscope (IRM) at an illumination wavelength near the exciton energy as a means to directly access the CDP in operating MoS_2_ capacitors and, more importantly, in MoS_2_ FETs. We call the technique excitonic reflection microscopy (XRM). We demonstrate how it can be used to reveal, with very strong contrast, the electron density gradient from source to drain in a biased transistor channel. We show that videos acquired while varying *V*_GS_ and *V*_DS_ give access to the CDP evolution with subsecond throughput, allowing direct observation of the gate vs drain competition in fixing the charge at each position in the channel. Effects such as resistive delays at grain boundaries, as well as local variations in the conduction band energy, are revealed.

## Results and Discussion

### Principle and Experimental Setup

The IRM configuration consists of an inverted microscope geometry, a high numerical aperture (NA) oil immersion objective, a transparent glass coverslip as the substrate, and a carefully chosen set of bandpass filters. IRM has its roots in biology and is designed to maximize thin-film interferences present in the sample itself or in the space between sample and substrate.^15,16^ For 2D materials, IRM can significantly improve contrast when compared with transillumination, as was shown by different groups for graphene^17^ and 2D TMDs.^18^ It is also a useful platform for *in situ* chemical and electrochemical experiments with 2D materials, as we showed in previous work.^19,20^

In this work, 2D MoS_2_ was grown by chemical vapor deposition (CVD) on SiO_2_/Si substrates following the same protocol as in^21^ (see Methods). A polymer-based method was used to transfer MoS_2_ to the borosilicate glass coverslips, where microfabrication steps took place both before and after transfer. Thorough cleaning was done to ensure that all polymer residue was removed (see section S7 in the Supporting Information). Thanks to the inverted geometry, an electrolyte can be added at the top interface with an immersed counter electrode, forming a first terminal. Second (and third) terminals are contacted directly to MoS_2_, resulting in either a capacitor or a FET.

**Figure 1:**
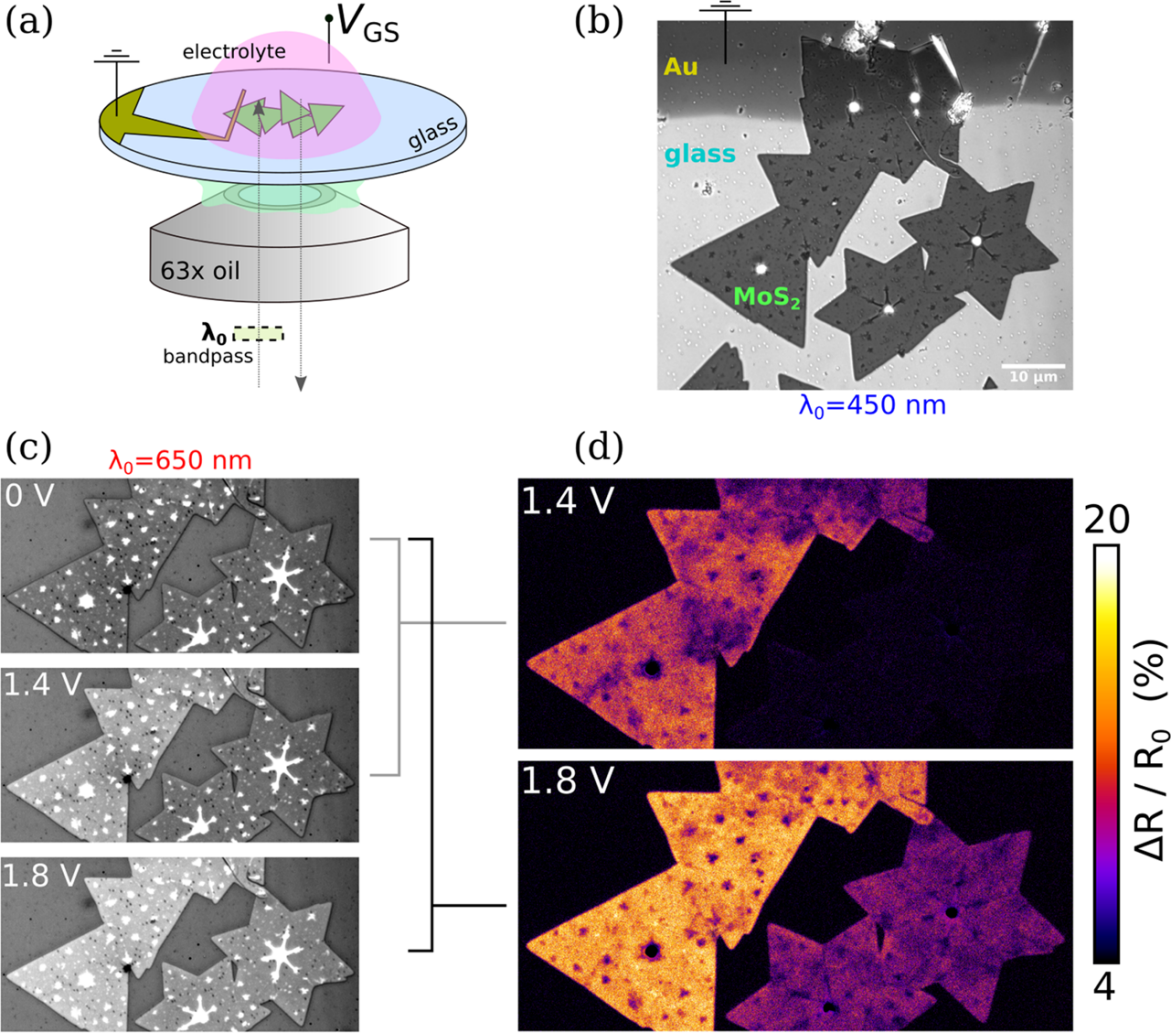
(a) Schematic of interference reflection microscope (IRM) with a CVD MoS_2_ electrolytic capacitor as the system under study. (b) IRM 8-bit micrograph at λ_0_ = 450 nm (grayscale range 32–171) in air, showing a strong negative contrast of *C* ≈ −46%. (c) Micrographs at λ_0_ = 650 nm (range 46–70) in the presence of an electrolyte (H_2_O/NaCl at 10^–1^ M) and for different applied gate voltages: 0, 1.4, and 1.8 V. (d) False color images of percent change in reflected light, at 1.4 V (upper panel) and 1.8 V (lower panel) using the 0 V image in (c) as reference. Images in (d) directly map the electron density evolution from 0 to 1.4 V and from 0 to 1.8 V, respectively.

Figure 1a shows a schematic of the capacitive case, with an IRM micrograph in Figure 1b showing a sample in air, prior to adding the electrolyte. The micrograph was taken using a bandpass filter centered at λ_0_ = 450 nm (10 nm bandwidth). It is well-known that the contrast of 2D materials is extremely sensitive to substrate type, observation geometry, and wavelength. In our observation conditions (on glass, in reflection, from the backside, and with air as the top medium), λ_0_ = 450 nm maximizes the contrast of MoS_2_ monolayers vs glass. Using the definition of contrast *C* ≡ (*I*_sample_/*I*_substrate_ – 1) × 100%, we found the monolayer contrast to be *C* ≈ −46%. (Here, *I*_sample_ and *I*_substrate_ are the reflected light intensity as measured by an 8-bit monochrome camera and thus on a 0–255 scale for each bit, for the sample and substrate, respectively.) With such high visibility and at this particular wavelength, various topographic details are readily observable: the growth seeds in the center of the crystalline domains appear as bright protrusions, while grain boundaries, adlayers, and local defects appear darker than the monolayer. When the electrolyte is added, the interference conditions change due to the higher refractive index of the electrolyte compared to air. In Figure 1, the electrolyte used is H_2_O/NaCl (10^–1^ M) with *n* = 1.33, which reverses the sign of the contrast, resulting in *C* ≈ +18%, for λ_0_ = 450 nm. (The visibility with electrolyte can in fact be improved, when the electrolyte has an even higher refractive index, which is the case for the diethylmethyl(2-methoxyethyl)ammonium bis(trifluoromethylsulfonyl)imide (DEME-TFSI) electrolyte (*n* = 1.4–1.5^22^) used later in the text, which results in *C* ≈ +98% at λ_0_ = 450 nm.)

For the CDP imaging mode (XRM), we use a bandpass filter centered at λ_0_ = 650 nm (10 nm bandwidth), which is near the A exciton of MoS_2_.^8^ Being a n-type semiconductor, its electron density *q* increases, when a positive bias is applied on the gate electrode (accumulation mode).^23−25^Figure 1c shows an example of such a charging experiment, with three wide-field reflectance images of the MoS_2_ (in 8-bit grayscale) at gate potentials of 0, 1.4, and 1.8 V. First note that with the electrolyte, at λ_0_ = 650 nm and *V*_GS_ = 0, adlayers now appear brighter than the monolayer, the opposite of the λ_0_ = 450 nm air case.

Once a bias is applied and using *V*_GS_ = 0 as a reference image, the percent change of reflected intensity is computed for each pixel and plotted in false color, in Figure 1d. The false color images directly map the change in the local charge density at each bias. Upon inspection of the *V*_GS_ = 1.4 V and *V*_GS_ = 1.8 V color maps, various observations can be made. First, the resulting signal-to-noise ratio is very strong, with a percent change signal approaching 15% at 1.8 V in the brightest areas corresponding to the highest electron density. This is well above the noise level so that there is no need for FFT filtering or other postprocessing of the data, conversely to some previous studies.^12,13^ Second, not all MoS_2_ crystallites react in the same way. It is striking that at 1.4 V, only part of the network of interconnected flakes is charged, while at 1.8 V all the connected flakes are revealed but with significant differences from flake to flake. Third, there are also inhomogeneities in the CDP at the scale of individual flakes (particularly observable at 1.4 V). There is thus a rich amount of information that can be garnered through the XRM modality, the results of which are further elaborated in the next section. The signal-to-noise ratio of the charge images are of sufficient quality down to around 500 ms throughput in the setup. This allows for acquisition of videos at 2 frames per second (fps), in wide-field mode, which is enough to capture some of the dynamics of the charging processes.

### Capacitive Charging Dynamics

**Figure 2:**
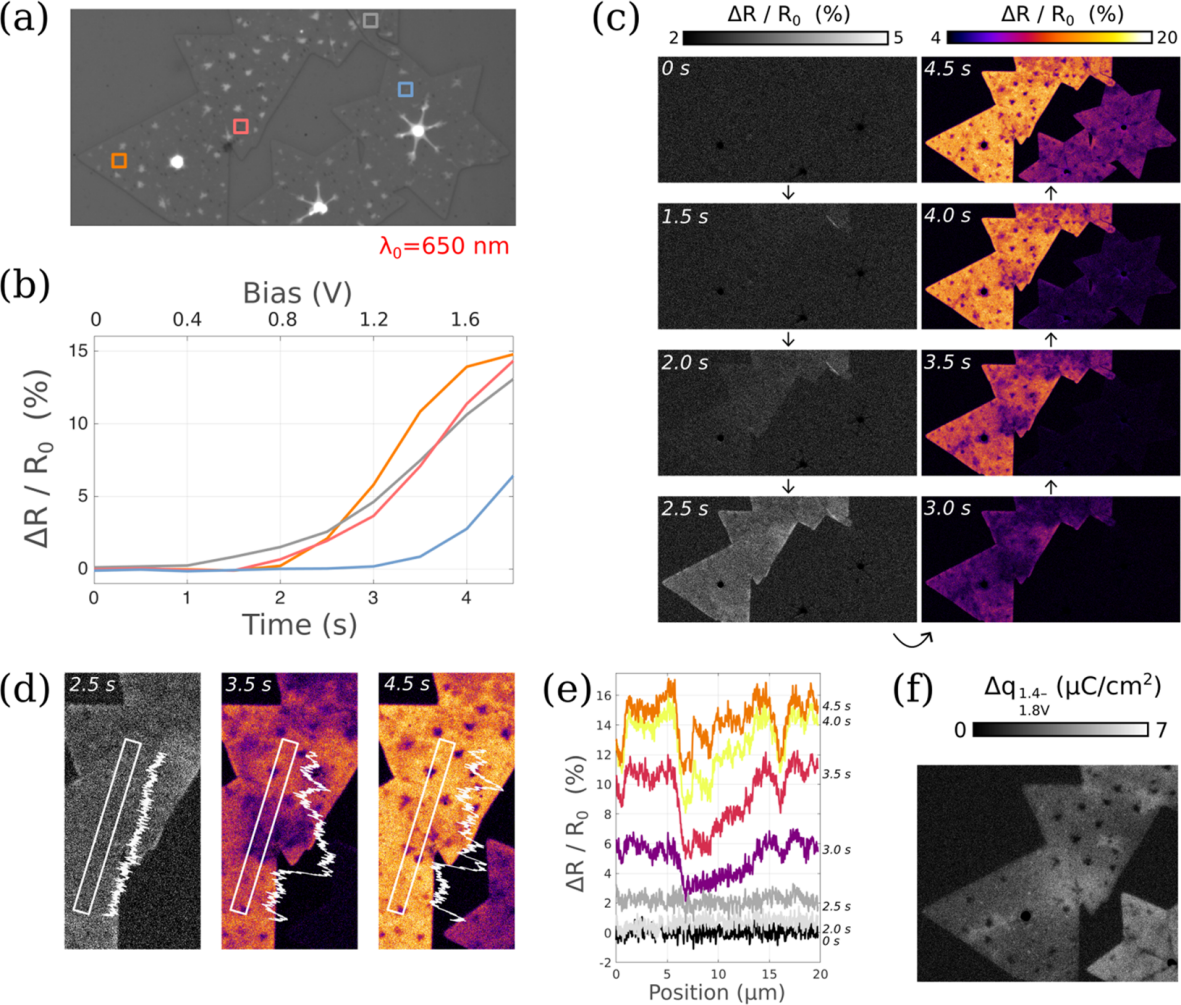
(a) IRM 8-bit micrograph (0–150 range) at λ_0_ = 650 nm in H_2_O/NaCl (10^–1^ M), with ROIs indicated as colored rectangles. (b) Evolution of ROI reflectance changes vs time and bias (*V*_GS_). (c) Film reel extracted from the video, as percent change of the *t* = −0.5 s frame. The color bars have been adjusted separately for the first and second half of the reel to better visualize the initial charging (*t* ≤ 2.5 s). (d) Averaged line profiles at frames *t* = 2.5, 3.5, 4.5 s. (e) Plotted profiles including additional frames. (f) CDP image showing the charge evolution in the 3.5–4.5 s interval, using charge sensitivity α = 2.0 × 10^4^ cm^2^C^–1^.

To capture the dynamics of electrolytic capacitive charging of the system shown in Figure 1b, *V*_GS_ was ramped at a rate of +400 mV/s while recording a video at λ_0_ = 650 nm and 2 fps. The *t* = 0 s (*V*_GS_ = 0 V) frame of the video is shown in Figure 2a, with various regions of interest (ROI) defined as shown by the colored rectangles. The signal was averaged for each ROI for each frame and then plotted as a function of time (bottom axis) and bias (top axis) in Figure 2b, as a percent change of the *t* = −0.5 s frame.

The first most evident result of the experiment is that not all of the interconnected MoS_2_ crystallites are being charged at the same rate. The network of MoS_2_ crystals on the left reacts first, while the network on the right reacts with an ∼1.5 s delay. This is evident both from the frame-by-frame reel in Figure 2c as well as from the orange vs blue ROI curves in Figure 2b. (Note that the color bars in Figure 2c have been adjusted separately for the first and second half of the reel, to better visualize the initial charging at ≤2.5 s.)

The likely explanation for this delay is that the node at the upper star-shaped MoS_2_ is highly resistive, which delays the charging of this effective RC circuit. The role of intra- and interflake resistances in the charging dynamics is further supported by the gray ROI which is close to the electrode, where it can be seen from its curve in Figure 2b that it is reacting first of all the ROIs, at the *t* = 1.5 s frame. In subsequent frames *t* = 2.0 and *t* = 2.5 s, the middle and bottom triangular crystallites are also charging at slightly different rates, most strikingly seen at *t* = 2.0 s, and also evidenced by comparing the gray, pink, and orange ROI curves, which start to increase in that order. This effect is a combination of inter- and intraflake resistances, with the former being the dominant one.

In Figure 2d, averaged line profiles are shown across part of the triangular crystallites, with additional profiles plotted in Figure 2e. Upon investigation, it is clear that at *t* = 3.5 s (1.4 V), there is a “valley” in the charge density, which is not present at *t* = 2.5 s, and then has been diminished at *t* = 4.5 s (1.8 V). It is likely that at the 1.4 V frame, the Fermi level is near the conduction band (*E*_c_) edge where such charge inhomogeneities would be more highly contrasted. Charge inhomogeneities are frequent in 2D materials and notably result from trapped charges in the substrate, and adsorbates, which create μm-sized variations in the 2D material’s *E*_c_.^26,27^ The same is supported by the pink ROI (located in the “valley”) which at 1.8 V has caught up to the orange ROI while being offset in prior time frames.

Up to here, the presented images corresponded to a reflectivity change. The correlation between percent change of reflectivity and a change in charge density can be modeled empirically according to the following proposed by Zhu et al.^12^ Δ*R*/*R*_0_ = αΔ*q* (where α is a constant of proportionality with units cm^2^ C^–1^). The relation Δ*q* = *C*_total_Δ*V* is used to estimate the change in charge density from the change in applied voltage (see section 1 in the Supporting Information for a more detailed discussion). However, the linear relationship with a constant α is applicable only in certain regimes of voltage change, as discussed later. An estimate of the Δ*R*/*R*_0_ to Δ*q* relationship was made and used to compute the Δ*q* image for the charging window 3.5–4.5 s (1.4–1.8 V), shown in Figure 2f. To estimate this relationship, the α parameter was determined using the orange ROI which is situated on a portion of the MoS_2_ network that is smooth, stable, and reacting in sync with the bias. Using a capacitance of *C*_EDL_= 10 μF/cm^2^ for H_2_O/NaCl (at 10^–1^ M),^28^ α was found to be α = 2.0 × 10^4^ cm^2^ C^–1^ (see fitting in Figure S1a of the Supporting Information) so that the change in accumulated charge accounts for ∼3 × 10^13^ electrons/cm^2^. Note that, in this particular time window, the “valleys” described earlier appear brighter in Δ*q* image Figure 2f. Indeed, since the charge tends to get homogeneous in the high-accumulation regime, the initially depleted puddles experienced a stronger evolution in that charging window.

Thus, there are multiple ways to extract and analyze local *E*_c_ variations by XRM: Δ*R*/*R*_0_ color maps, averaged line profiles, and a quantitative estimate of the Δ*q* variations in a particular charging window. Such local variations in *E*_c_ of 2D materials have been the subject of intense study, often using Raman and photoluminescence/reflectance spectroscopies in scanning mode with ∼1 μm sized laser spots,^3^ or by scanning probe techniques like KPFM, MIM, and conducting AFM (CAFM). These methods offer certain advantages. Raman spectroscopy provides direct access to phonon energies (which correlate with charge density and strain), and PL provides exciton/trion energies, intensities, and line broadenings. KPFM provides direct access to the surface potential. In static mode, scans of CVD-grown TMDs can reveal variations due to topographies, like grain boundaries and adlayers in a clearly discernible manner,^29,30^ as well as external influences like humidity.^31^*In operando* experiments have also been performed with KPFM for MoS_2_ FETs,^32,33^ and with conducting AFM revealing the resistance of grain boundaries in MoS_2_.^34^ MIM scans during capacitive charging also have the capacity to reveal local conductance variations, where Wu et al. showed how the Fermi level near the *E*_c_ edge revealed variations with better contrast.^2^

With its subsecond throughput, XRM provides a complementary means to capture important charging mechanisms. The *E*_c_ valley shown was identified with only a few seconds of acquisition, and furthermore the high throughput revealed a charging delay in ∼50% of the active area, which would have been missed by conventional spectroscopies. Electrical measurements such as capacitance spectroscopy which have been used for MoS_2_^35^ require knowledge of the surface area. If the entire surface in Figure 2 was assumed to be active on the same time scales, there would have been a significant miscalculation when extracting physical parameters from the *C*(ω) response. This highlights the utility of XRM for determining the active regions in the local charge modulation time-frequency response landscape.

### Field-Effect Transistor

**Figure 3:**
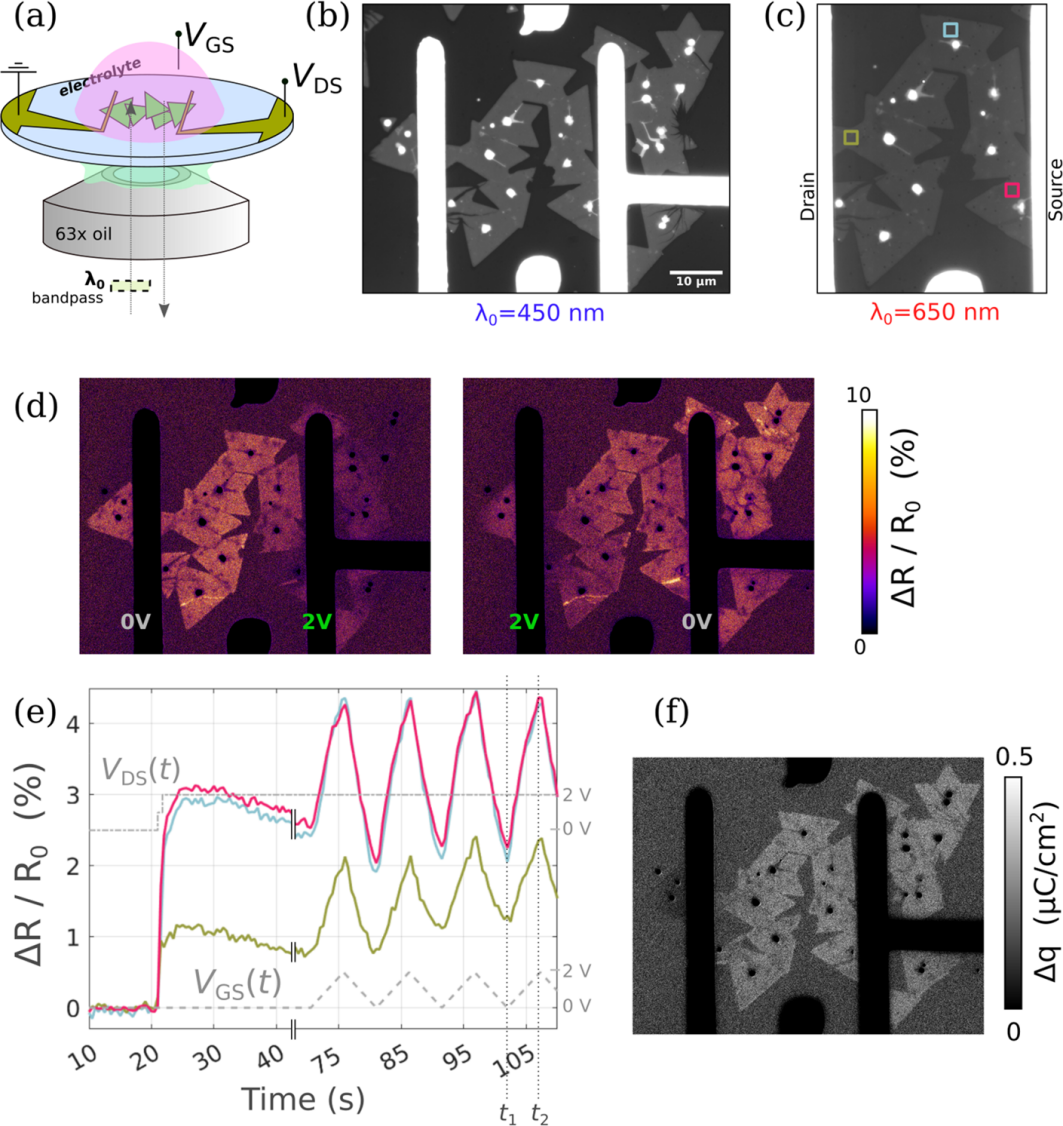
(a) Schematic of an IL-gated MoS_2_ FET coupled with IRM. (b) IRM 8-bit micrograph (0–200 range) at λ_0_ = 450 nm with electrolyte added, resulting in a high contrast *C* ≈ +98%. (c) λ_0_ = 650 nm (0–255 range) with ROIs indicated as colored rectangles for XRM. (d) Percent change reflectance images for *V*_DS_ = 2 V applied first on the right electrode (left panel) and then on the left electrode (right panel). (e) ROI reflectance changes as a function of time and bias. The applied *V*_DS_ and *V*_GS_ are indicated on the right *Y*-axis. (f) Δ*q* CDP computed for the time range *t*_1_ = 102 s to *t*_2_ = 107 s using α = 8.8 × 10^4^ cm^2^ C^–1^.

**Figure 4:**
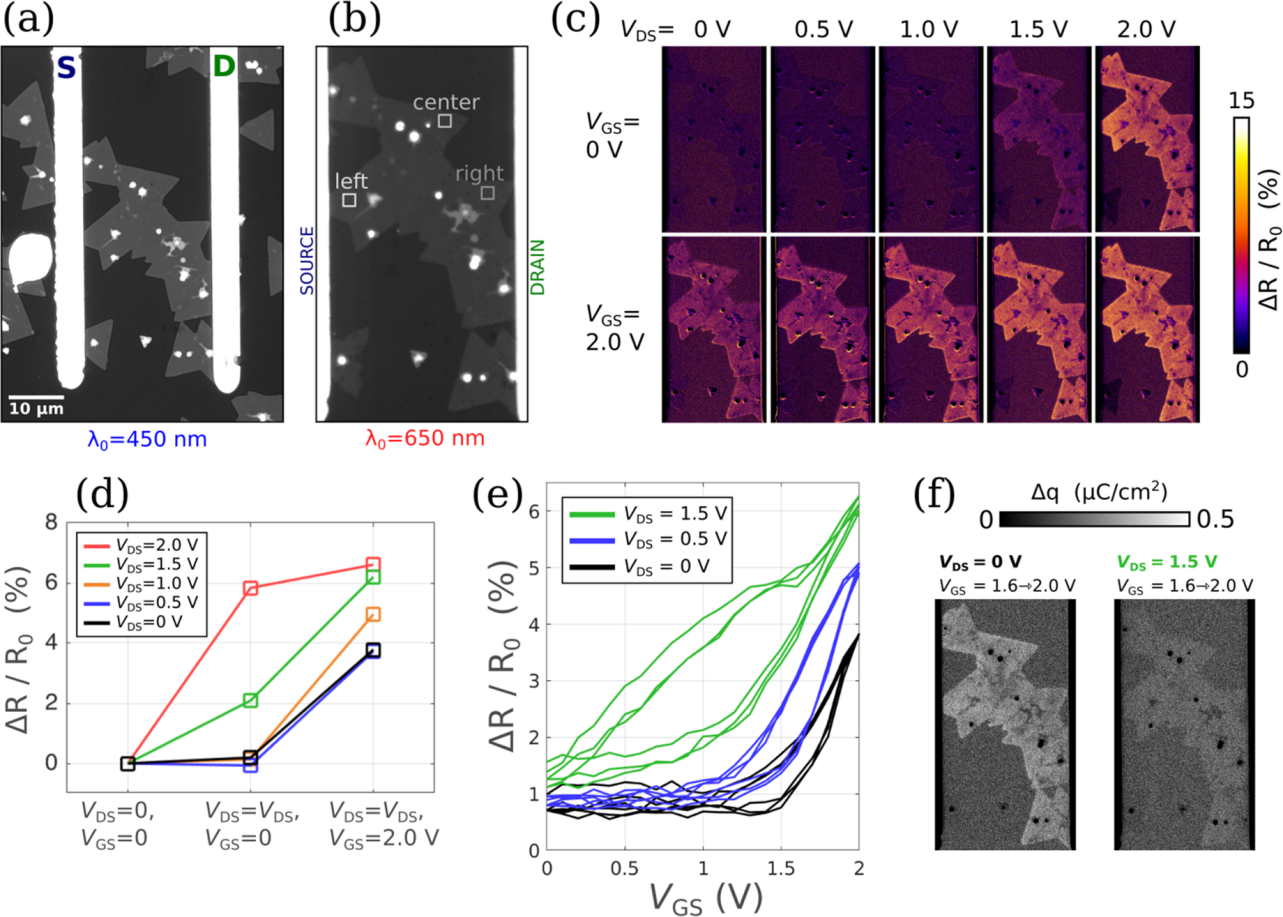
(a) IRM 8-bit micrograph (range 0–150) at λ_0_ = 450 nm with electrolyte, of an FET device, and (b) at λ_0_ = 650 nm (range 0–255) with 3 ROIs defined. (c) Effect of drain and gate biases on Δ*R*/*R*_0_ for drain values 0, 0.5, 1.0, 1.5, 2.0 V and gate values of 0 V (upper row) and then 2.0 V (lower row), with (d) the corresponding plot for the ROI defined at the center of the channel. (e) Reflectance change vs gate voltage. (f) Charge density images generated using α = 8.8 × 10^4^ cm^2^ C^–1^ in the *V*_GS_ window 1.6–2.0 V.

Having established the potential of the XRM technique to study charging effects in MoS_2_ capacitors, we now turn to its use in the more complex case of 3-terminal devices. To that end, we first present (Figure 3) the principle of the experiment and the different ways of exploiting XRM data in this context. Then (in Figure 4) we take benefit of the dual way of using XRM (static charge mapping and time-dependent local charge evolution measurement) to extract relevant information from transistor operation.

First, in order to establish the capability of XRM to resolve the CDP in MoS_2_ FETs, ionic liquid (IL) gated devices were realized, in the configuration shown in Figure 3a. A micrograph taken at λ_0_ = 450 nm is shown in Figure 3b, which provides the best contrast with the IL added (*C* ≈ +98%). A micrograph at λ_0_ = 650 nm, for the XRM mode, is shown in Figure 3c with three ROIs defined as colored rectangles (including close to the source, close to the drain, and in the central part of the channel). The channel consists of interconnected crystalline monolayer MoS_2_ domains separated by grain boundaries.

The gate electrolyte used in this section was DEME-TFSI, an ionic liquid commonly used in the literature for MoS_2_ FETs.^36−39^ While this ionic liquid has a lower electrical double-layer capacitance (*C*_EDL_) than the H_2_O/NaCl used for capacitors in the previous section, its use here significantly improves device stability upon cycling, which is important, as elaborated below. We estimated the gate capacitance in this configuration to be *C*_EDL_ = 0.8 μF/cm^2^ in a different experiment by comparing the transfer characteristics of a transistor controlled successively by an electrolytic gate and a conventional Si/SiO_2_ back-gate (see the Supporting Information and Figure S2 for details). As a first test, a drain bias of 2 V was applied on the right electrode with the source and gate held at 0 V. The same was then repeated with the drain and source swapped. The percent change in reflectivity was computed for both cases and is shown in Figure 3d. We clearly observe a strong charge accumulation in the channel associated with a significant charge gradient from the source to drain in a decreasing fashion. This is the expected behavior for a Schottky-barrier FET in the ON-state. Note that while *V*_GS_ = 0 in this particular experiment, the use of a high drain bias (2 V) is sufficient to turn the transistor ON, as confirmed by separated electrical measurements using the same electrolytic gate (Figure S3) and further discussed with Figure 4.

To further illustrate the technique, a video was recorded at 2 fps where a drain bias of 2 V is applied at *t* = 20 s and held constant, while the gate was swept multiple times between 0 and 2 V at 400 mV/s starting at *t* = 70 s. The reflectance change extracted from the 3 ROIs indicated in Figure 3c is plotted as a function of time in Figure 3e using the *t* = 11 s frame as the reference *R*_0_. (Also, the right-most image of Figure 3d corresponds to ΔR/R_0_[@*t* = 22.5 s] of this video.) The plot first confirms the formation of a charge density gradient from source to drain when *V*_DS_ = 2 V is initially applied (the red curve corresponding to a 3 times stronger contrast compared to the green curve). It also indicates that the charge density in the center of the channel (blue curve) is mostly following the one on the source side, as expected in such a FET in the ON-state. Then, once the gate bias is swept, the contrast is clearly modulated. This way of extracting data from an XRM experiment allows directly assessing (i) the respective efficiency of *V*_DS_ and *V*_GS_ in fixing the charge density at any point along the channel, (ii) the stability of the charging/discharging upon multiple *V*_GS_ sweep cycles, and (iii) the evolution of the charge density when both *V*_DS_ and *V*_GS_ are kept constant. For example, one can observe a slow decay of the charge density in the 25–65 s time range. This expected behavior is similar to the decay of the ON-state current observed in electrical measurements at fixed bias for all types of nanomaterial-based FETs. It is related to the progressive trapping of charges either in defects of the material channel itself, or in the gate dielectric or in adsorbed molecules. Such charge trapping leads to the commonly observed hysteresis in the *I*_D_(*V*_GS_) characteristics present in virtually all MoS_2_-FET studies,^40−42^ when MoS_2_ lies on SiO_2_ or other dielectrics with a significant density of shallow trap states. Finally, the third way of extracting data from XRM videos consists of computing Δ*q* maps in particular bias ranges. Figure 3f displays such a Δ*q* map extracted from one of the gate sweeps (0 → 2 V) between *t*_1_ = 102 s and *t*_2_ = 107 s (marked with dotted lines in Figure 3e) obtained using an α value of α = 8.8 × 10^4^ cm^2^ C^–1^ (this evaluation of α is discussed hereafter with Figure 4). Based on this, the near-drain (green) ROI experiences a Δ*q* of ∼0.12 μC/cm^2^ in this time range, while the channel center (blue) and near-source (red) both experience Δ*q* of ∼0.24 μC/cm^2^ (∼1.5 × 10^12^ electrons/cm^2^).

In Figure 3, a 2 V source–drain bias was used for clarity due to its strong impact on the reflectivity. It is, however, an unconventionally high *V*_DS_, able to turn ON the device even at *V*_GS_ = 0. FETs are more commonly operated at low to moderate *V*_DS_ so that the gate bias dominates the control of the charge in the channel. Thus, the next portion will focus on XRM across a range of *V*_DS_, and how it can be used to directly visualize this *V*_DS_, *V*_GS_ competition for control of the channel charge.

To that end, a second FET device was realized, shown in Figure 4a at λ_0_ = 450 nm with high contrast. Figure 4b is taken at λ_0_ = 650 nm, the compatible wavelength for XRM condition, with 3 ROIs defined, situated near the source, near the channel center, and near the drain. Voltages of *V*_DS_ = 0, 0.5, 1.0, 1.5, and 2.0 V were explored for the drain. For the gate, a maximum of *V*_GS_ = 2 V was applied for each case. Micrographs of reflectivity change corresponding to these ten (*V*_DS_, *V*_GS_) conditions are shown in Figure 4c. The *V*_DS_ = 0 to *V*_DS_ = 1 V cases correspond to typical n-type FET operation with little charge in the channel for *V*_GS_ = 0 and strong electron accumulation observed at *V*_GS_ = 2 V. Increasing *V*_DS_ to 1.5 and eventually 2 V induces electron accumulation even at *V*_GS_ = 0 (as discussed earlier for Figure 3). Unsurprisingly, combining *V*_DS_ = 2 V and *V*_GS_ = 2 V leads to the maximum charge density in the channel. This is in close agreement with typical electrical measurements of MoS_2_–FETs using such an ionic liquid as a gate electrolyte, as exemplified in Figure S3.

To quantify this balance of *V*_DS_ vs *V*_GS_ efficiency in fixing the charge, the responses at the center ROI are plotted in Figure 4d. The other ROIs along with other data are shown in Figure S4 of the Supporting Information. The ability to isolate any ROI along the channel is of significant utility for XRM, allowing for CDP analysis at any (*V*_DS_, *V*_GS_) polarization point and any position along the channel.

Next, the results of the videos taken at *V*_DS_ = 0, 0.5, and 1.5 V while the gate was swept back and forth from charge depletion (*V*_GS_ = 0 V) to charge accumulation (*V*_GS_ = 2 V), at a rate of 200 mV/s, are presented. The reflectance change is plotted as a function of time in Figure S4b and as a function of *V*_GS_ in Figure 4e. Interestingly, these optical Δ*R*/*R*_0_(*V*_GS_) curves bear strong similarities with conventional electrical transfer characteristics *I*_D_(*V*_GS_). In particular, they both allow extracting the gate efficiency above the threshold voltage (in the form of a transconductance for electrical measurements, or here, in the form of a “*trans-reflectance*”) and to assess the level of trapped-charge related hysteresis, when alternating the gate sweep direction. (A more detailed analysis of the hysteresis effect by XRM is presented in Figures S9 and S10 in the Supporting Information. In particular, Figure S9 shows that the width of the hysteresis loop at each *V*_DS_ can be measured and converted into charge density similarly to hysteresis analysis in electrical measurements.) Furthermore, XRM allows mapping the hysteresis effect at any (*V*_GS_, *V*_DS_) polarization point, as exemplified by the maps presented in Figure S10. Yet, reflectivity intrinsically contains two additional features: (i) since charge is measured in place of current, data can be collected at *V*_DS_ = 0 V, in the absence of current flow, and (ii) in place of a global current as the only observable, reflectivity is measured locally so that inhomogeneties in the channel are directly mapped. As already observed in the case of capacitors in Figure 2, not all the flakes are identically well connected, so that the effective channel can significantly differ from the geometrically defined one.

Finally, using the *V*_DS_ = 0 case (black curve in Figure 4e) as a reference, the α parameter was fitted and found to be α = 8.8 × 10^4^ cm^2^ C^–1^ (Figure S1b). Using this, CDP images were generated and are presented in Figure 4f for the gate bias range 1.6–2.0 V (time range 38.5–40.5 s) for the case of *V*_DS_ = 0 (left) and *V*_DS_ = 1.5 V (right). They indicate that the gate is altering the CDP to a lesser extent at *V*_DS_ = 1.5 V when compared with *V*_DS_ = 0 V, in that regime. This is in agreement with the increased impact of the drain on fixing the charge when the transistor is operated at high *V*_DS_. Note that the left map of Figure 4f (obtained at *V*_DS_ = 0) corresponds to a gate bias swing starting at 1.6 V. This bias also corresponds to the threshold voltage of the reflectivity change (as seen from the black curve in Figure 4e). It is thus reasonable to consider this Δ*q* map to be simply a *q* map (the charge at *V*_TH_ being negligible compared to the charge in the ON-state).

As one of the main assets of XRM is its capability to map charge density evolution along the channel, one important aspect concerns the size of the ROI that is required to correctly extract device parameters. To explore this point, we calculated Δ*R*/*R*_0_ for a range of ROIs that vary in size but are centered at the same point, where a gate was swept back and forth to capacitively charge and discharge the monolayer MoS_2_. The time-dependent traces were thus plotted for 6 ROIs that vary from 50 × 50 pixels (∼1.47 × 1.47 μm^2^) down to 4 × 4 pixels (∼0.12 × 0.12 μm^2^), shown in Figure S5. We found that the gate modulation effect is still easily distinguishable from the noise even down to the smallest ROI, while 10 × 10 pixels (∼0.29 × 0.29 μm^2^) can be considered the lower limit of usable signal to extract the “trans-reflectance” mentioned earlier.

Finally, to confirm that the measured signal indeed corresponds to the previously mentioned excitonic effect, we measured the IRM response for *V*_GS_ = 2 V using 12 bandpass filters ranging from λ_0_ = 400 nm to λ_0_ = 700 nm. The result is shown in Figure S6 and clearly indicates that significant modulation takes place near excitonic wavelengths (590–660 nm), while other wavelengths show a minimal or nonexistent change.

## Conclusion

XRM proves to be a powerful tool for studying MoS_2_ devices and, in particular, field-effect transistors. The ability to map charge modulation in wide-field mode with subsecond throughput provides multiple avenues to study 2D FETs *in operando* (see Table S11 in the Supporting Information for a comparison with other techniques). First, the drain vs gate competition over the channel potential profile is shown, which is central in determining the maximum attainable subthreshold swing.^43^ Second, the capability to study the differing response of crystallites in a multicrystalline channel is shown, an aspect relevant both in capacitive devices like modulators^6^ and 2D redox electrodes^44,45^ as well as for transistors, where the active surface area is critical when calculating performance metrics. We showed that XRM’s throughput was capable of revealing areas of different resistive coupling operating at different time scales. Third, channel inhomogeneities and their effect on charge distribution play a key role in FET-based sensors, where often, resistive grain boundaries are leveraged by design to improve sensitivity to certain analytes.^7,46−48^ Grain boundaries, edges, and adlayers also play an important role in redox electrodes,^38^ where such features may provide increased electrochemical activity. Additional data analyzing charging at grain boundaries is presented in Figure S8 of the Supporting Information.

In this work, we used XRM for the study of MoS_2_. The same principle can be applied to other TMDs as well, including WS_2_ and MoSe_2_, which also exhibit a high sensitivity of their optical properties to the charge density.^9,49,50,11^ In fact, the modulation depths have been reported to be superior to that of MoS_2_. Furthermore, Scuri et al.^11^ detected changes in reflectivity via wide-field images upon charging MoSe_2_, using a conventional upright microscope on a SiO_2_/Si substrate, indicating that configurations other than IRM could be used, albeit with lower lateral resolution and reduced interference contrast. For other TMDs, an appropriate wavelength must be chosen in each case to match the exciton energies where the correlation is most present. Finally, the technique will show its full potential for devices based on van der Waals heterostructures,^51^ where the use of different wavelengths will allow mapping charge transfer in complex configurations. With its relatively simple design and low cost, XRM will undoubtedly become a tool of choice for 2D materials research in combination with conventional techniques.

## Methods

### Sample Preparation

MoS_2_ domains were synthesized by chemical vapor deposition (CVD), with the conditions described in ref (21). Sulfur and MoO_3_ powders were annealed in a nitrogen flow within the quartz tube of a tubular furnace using their respective positions to adjust their temperature (in the 180–220 and 700–750 °C ranges for S and MoO_3_, respectively). The Si/SiO_2_ substrate, covered with a thin film of perylene-3,4,9,10-tetracarboxylic acid tetrapotassium salt (PTAS) acting as seed promoter, was placed at the same temperature as the MoO_3_ source and kept at the maximal temperature for 10 min.

### Camera and Settings

All images and videos were acquired using an IDS UI-3880CP-M-GL Rev.2 (AB00854) monochrome 1.8 in. CMOS camera. In the uEye Cockpit software, the “raw8” format was selected so that no gamma power function or other processing was applied to the acquisitions. In this configuration, the 8-bit grayscale images have a linear response to the light intensity.

## Supporting Information Available

Details on fitting of α parameter and extraction of DEME-TFSI capacitance, electrical characteristic of DEME-TFSI gated MoS_2_ FET, additional data for Figure 4, analysis of effect of ROI size, reflectivity change measured at 12 wavelengths, details of cleaning after transfer, additional data on charging at grain boundaries, hysteresis width evaluations from reflectivity plots, hysteresis charge maps at different biases, and comparison of XRM with other techniques (PDF)

## References

[ref1] Matsunaga M.; Higuchi A.; He G.; Yamada T.; Krüger P.; Ochiai Y.; Gong Y.; Vajtai R.; Ajayan P. M.; Bird J. P.; Aoki N. Nanoscale-Barrier Formation Induced by Low-Dose Electron-Beam Exposure in Ultrathin MoS_2_ Transistors. ACS Nano 2016, 10 (10), 9730–9737. 10.1021/acsnano.6b05952. 27704777

[ref2] Wu D.; Li X.; Luan L.; Wu X.; Li W.; Yogeesh M. N.; Ghosh R.; Chu Z.; Akinwande D.; Niu Q.; Lai K. Uncovering Edge States and Electrical Inhomogeneity in MoS_2_ Field-Effect Transistors. Proc. Natl. Acad. Sci. U.S.A. 2016, 113 (31), 8583–8588. 10.1073/pnas.1605982113. 27444021 PMC4978287

[ref3] Michail A.; Delikoukos N.; Parthenios J.; Galiotis C.; Papagelis K. Optical Detection of Strain and Doping Inhomogeneities in Single Layer MoS_2_. Appl. Phys. Lett. 2016, 108 (17), 173102 10.1063/1.4948357.

[ref4] Fiori G.; Bonaccorso F.; Iannaccone G.; Palacios T.; Neumaier D.; Seabaugh A.; Banerjee S. K.; Colombo L. Electronics Based on Two-Dimensional Materials. Nat. Nanotechnol. 2014, 9 (10), 768–779. 10.1038/nnano.2014.207. 25286272

[ref5] Hwangbo S.; Hu L.; Hoang A. T.; Choi J. Y.; Ahn J.-H. Wafer-Scale Monolithic Integration of Full-Colour Micro-LED Display Using MoS_2_ Transistor. Nat. Nanotechnol. 2022, 17 (5), 500–506. 10.1038/s41565-022-01102-7. 35379943

[ref6] Datta I.; Chae S. H.; Bhatt G. R.; Tadayon M. A.; Li B.; Yu Y.; Park C.; Park J.; Cao L.; Basov D. N.; Hone J.; Lipson M. Low-Loss Composite Photonic Platform Based on 2D Semiconductor Monolayers. Nat. Photonics 2020, 14 (4), 256–262. 10.1038/s41566-020-0590-4.

[ref7] Hayashi K.; Kataoka M.; Jippo H.; Yamaguchi J.; Ohfuchi M.; Sato S. Highly Sensitive NO2 Detection by TVS-Grown Multilayer MoS_2_ Films. ACS Omega 2022, 7 (2), 1851–1860. 10.1021/acsomega.1c05113. 35071877 PMC8771694

[ref8] Mak K. F.; He K.; Lee C.; Lee G. H.; Hone J.; Heinz T. F.; Shan J. Tightly Bound Trions in Monolayer MoS_2_. Nat. Mater. 2013, 12 (3), 207–211. 10.1038/nmat3505. 23202371

[ref9] Yu Y.; Yu Y.; Huang L.; Peng H.; Xiong L.; Cao L. Giant Gating Tunability of Optical Refractive Index in Transition Metal Dichalcogenide Monolayers. Nano Lett. 2017, 17 (6), 3613–3618. 10.1021/acs.nanolett.7b00768. 28505462

[ref10] Kravets V. G.; Wu F.; Auton G. H.; Yu T.; Imaizumi S.; Grigorenko A. N. Measurements of Electrically Tunable Refractive Index of MoS_2_ Monolayer and Its Usage in Optical Modulators. npj 2D Mater. Appl. 2019, 3, 36 10.1038/s41699-019-0119-1.

[ref11] Scuri G.; Zhou Y.; High A. A.; Wild D. S.; Shu C.; De Greve K.; Jauregui L. A.; Taniguchi T.; Watanabe K.; Kim P.; Lukin M. D.; Park H. Large Excitonic Reflectivity of Monolayer MoSe_2_ Encapsulated in Hexagonal Boron Nitride. Phys. Rev. Lett. 2018, 120 (3), 037402 10.1103/PhysRevLett.120.037402. 29400519

[ref12] Zhu H.; Zhang F.; Wang H.; Lu Z.; Chen H.; Li J.; Tao N. Optical Imaging of Charges with Atomically Thin Molybdenum Disulfide. ACS Nano 2019, 13 (2), 2298–2306. 10.1021/acsnano.8b09010. 30636406

[ref13] Zhu H.; Chen Z.; Chen Y.; Zhu J. Affinities and Kinetics Detection of Protein-Small Molecule Interactions with a Monolayer MoS_2_ Based Optical Imaging Platform. Small 2022, 18 (29), 2202622 10.1002/smll.202202622. 35726050

[ref14] Zhao X.; Zhou X.-L.; Yang S.-Y.; Min Y.; Chen J.-J.; Liu X.-W. Plasmonic Imaging of the Layer-Dependent Electrocatalytic Activity of Two-Dimensional Catalysts. Nat. Commun. 2022, 13, 7869 10.1038/s41467-022-35633-3. 36550149 PMC9780338

[ref15] Curtis A. S. G. The Mechanism of Adhesion of Cells to Glass: A Study by Interference Reflection Microscopy. J. Cell Biol. 1964, 20 (2), 199–215. 10.1083/jcb.20.2.199. 14126869 PMC2106393

[ref16] Limozin L.; Sengupta K. Quantitative Reflection Interference Contrast Microscopy (RICM) in Soft Matter and Cell Adhesion. ChemPhysChem 2009, 10 (16), 2752–2768. 10.1002/cphc.200900601. 19816893

[ref17] Li W.; Moon S.; Wojcik M.; Xu K. Direct Optical Visualization of Graphene and Its Nanoscale Defects on Transparent Substrates. Nano Lett. 2016, 16 (8), 5027–5031. 10.1021/acs.nanolett.6b01804. 27351749

[ref18] Goggin D. M.; Zhang H.; Miller E. M.; Samaniuk J. R. Interference Provides Clarity: Direct Observation of 2D Materials at Fluid-Fluid Interfaces. ACS Nano 2020, 14 (1), 777–790. 10.1021/acsnano.9b07776. 31820924

[ref19] Campidelli S.; Abou Khachfe R.; Jaouen K.; Monteiller J.; Amra C.; Zerrad M.; Cornut R.; Derycke V.; Ausserré D. Backside Absorbing Layer Microscopy: Watching Graphene Chemistry. Sci. Adv. 2017, 3 (5), e1601724 10.1126/sciadv.1601724. 28508053 PMC5429035

[ref20] Jaouen K.; Cornut R.; Ausserré D.; Campidelli S.; Derycke V. Ideal Optical Contrast for 2D Material Observation Using Bi-Layer Antireflection Absorbing Substrates. Nanoscale 2019, 11 (13), 6129–6135. 10.1039/C8NR09983A. 30869677

[ref21] Arrighi A.; Ullberg N.; Derycke V.; Grévin B. A Simple KPFM-Based Approach for Electrostatic- Free Topographic Measurements: The Case of MoS_2_ on SiO_2_. Nanotechnology 2023, 34 (21), 215705 10.1088/1361-6528/acbe02. 36812541

[ref22] Xu G.; Lu R.; Liu J.; Chiu H.-Y.; Hui R.; Wu J. Z. Photodetection Based on Ionic Liquid Gated Plasmonic Ag Nanoparticle/Graphene Nanohybrid Field Effect Transistors. Adv. Opt. Mater. 2014, 2 (8), 729–736. 10.1002/adom.201400077.

[ref23] Radisavljevic B.; Radenovic A.; Brivio J.; Giacometti V.; Kis A. Single-Layer MoS_2_ Transistors. Nat. Nanotechnol. 2011, 6 (3), 147–150. 10.1038/nnano.2010.279. 21278752

[ref24] Liu H.; Neal A. T.; Ye P. D. Channel Length Scaling of MoS_2_ MOSFETs. ACS Nano 2012, 6 (10), 8563–8569. 10.1021/nn303513c. 22957650

[ref25] Di Bartolomeo A.; Kumar A.; Durante O.; Sessa A.; Faella E.; Viscardi L.; Intonti K.; Giubileo F.; Martucciello N.; Romano P.; Sleziona S.; Schleberger M. Temperature-Dependent Photoconductivity in Two-Dimensional MoS_2_ Transistors. Materials Today Nano 2023, 24, 100382 10.1016/j.mtnano.2023.100382.

[ref26] Xin H.; Zhang J.; Yang C.; Chen Y. Direct Detection of Inhomogeneity in CVD-Grown 2D TMD Materials via K-Means Clustering Raman Analysis. Nanomaterials 2022, 12 (3), 414 10.3390/nano12030414. 35159759 PMC8840665

[ref27] Kolesnichenko P. V.; Zhang Q.; Yun T.; Zheng C.; Fuhrer M. S.; Davis J. A. Disentangling the Effects of Doping, Strain and Disorder in Monolayer WS_2_ by Optical Spectroscopy. 2D Mater. 2020, 7 (2), 025008 10.1088/2053-1583/ab626a.

[ref28] Khademi M.; Barz D. P. J. Structure of the Electrical Double Layer Revisited: Electrode Capacitance in Aqueous Solutions. Langmuir 2020, 36 (16), 4250–4260. 10.1021/acs.langmuir.0c00024. 32227968

[ref29] Precner M.; Polaković T.; Trainer D. J.; Putilov A. V.; Di Giorgio C.; Cone I.; Xi X. X.; Iavarone M.; Karapetrov G. Metastable Defects in Monolayer and Few-Layer Films of MoS_2_; AIP: 2018; Vol. 2005, p 020004 10.1063/1.5050721. PMC5928116 29712931

[ref30] Moore D.; Jo K.; Nguyen C.; Lou J.; Muratore C.; Jariwala D.; Glavin N. R. Uncovering Topographically Hidden Features in 2D MoSe_2_ with Correlated Potential and Optical Nanoprobes. npj 2D Mater. Appl. 2020, 4, 44 10.1038/s41699-020-00178-w.

[ref31] Feng Y.; Zhang K.; Li H.; Wang F.; Zhou B.; Fang M.; Wang W.; Wei J.; Wong H. S. P. In Situ Visualization and Detection of Surface Potential Variation of Mono and Multilayer MoS_2_ under Different Humidities Using Kelvin Probe Force Microscopy. Nanotechnology 2017, 28 (29), 295705 10.1088/1361-6528/aa7183. 28664874

[ref32] Vaknin Y.; Dagan R.; Rosenwaks Y. Schottky Barrier Height and Image Force Lowering in Monolayer MoS_2_ Field Effect Transistors. Nanomaterials 2020, 10 (12), 2346 10.3390/nano10122346. 33255993 PMC7761329

[ref33] Matković A.; Petritz A.; Schider G.; Krammer M.; Kratzer M.; Karner Petritz E.; Fian A.; Gold H.; Gärtner M.; Terfort A.; Teichert C.; Zojer E.; Zojer K.; Stadlober B. Interfacial Band Engineering of MoS_2_/Gold Interfaces Using Pyrimidine Containing Self Assembled Monolayers: Toward Contact Resistance Free Bottom Contacts. Adv. Electron. Mater. 2020, 6 (5), 2000110 10.1002/aelm.202000110.

[ref34] Giannazzo F.; Bosi M.; Fabbri F.; Schilirò E.; Greco G.; Roccaforte F. Direct Probing of Grain Boundary Resistance in Chemical Vapor Deposition Grown Monolayer MoS_2_ by Conductive Atomic Force Microscopy. Phys. Status Solidi RRL 2020, 14 (2), 1900393 10.1002/pssr.201900393.

[ref35] Zhao Y.; Tripathi M.; Čerņevičs K.; Avsar A.; Ji H. G.; Gonzalez Marin J. F.; Cheon C.-Y.; Wang Z.; Yazyev O. V.; Kis A. Electrical Spectroscopy of Defect States and Their Hybridization in Monolayer MoS_2_. Nat. Commun. 2023, 14, 44 10.1038/s41467-022-35651-1. 36596799 PMC9810731

[ref36] Wang F.; Stepanov P.; Gray M.; Lau C. N.; Itkis M. E.; Haddon R. C. Ionic Liquid Gating of Suspended MoS_2_ Field Effect Transistor Devices. Nano Lett. 2015, 15 (8), 5284–5288. 10.1021/acs.nanolett.5b01610. 26181777

[ref37] Zhang Y.; Ye J.; Matsuhashi Y.; Iwasa Y. Ambipolar MoS_2_ Thin Flake Transistors. Nano Lett. 2012, 12 (3), 1136–1140. 10.1021/nl2021575. 22276648

[ref38] Du J.; Ge C.; Riahi H.; Guo E.; He M.; Wang C.; Yang G.; Jin K. Dual Gated MoS_2_ Transistors for Synaptic and Programmable Logic Functions. Adv. Electron. Mater. 2020, 6 (5), 1901408 10.1002/aelm.201901408.

[ref39] Perera M. M.; Lin M.-W.; Chuang H.-J.; Chamlagain B. P.; Wang C.; Tan X.; Cheng M. M.-C.; Tománek D.; Zhou Z. Improved Carrier Mobility in Few-Layer MoS_2_ Field-Effect Transistors with Ionic-Liquid Gating. ACS Nano 2013, 7 (5), 4449–4458. 10.1021/nn401053g. 23590723

[ref40] Di Bartolomeo A.; Genovese L.; Giubileo F.; Iemmo L.; Luongo G.; Foller T.; Schleberger M. Hysteresis in the Transfer Characteristics of MoS_2_ Transistors. 2D Mater. 2018, 5 (1), 015014 10.1088/2053-1583/aa91a7.

[ref41] Shu J.; Wu G.; Guo Y.; Liu B.; Wei X.; Chen Q. The Intrinsic Origin of Hysteresis in MoS_2_ Field Effect Transistors. Nanoscale 2016, 8 (5), 3049–3056. 10.1039/C5NR07336G. 26782750

[ref42] Guo Y.; Wei X.; Shu J.; Liu B.; Yin J.; Guan C.; Han Y.; Gao S.; Chen Q. Charge Trapping at the MoS_2_-SiO_2_ Interface and Its Effects on the Characteristics of MoS_2_ Metal-Oxide-Semiconductor Field Effect Transistors. Appl. Phys. Lett. 2015, 106 (10), 103109 10.1063/1.4914968.

[ref43] Yoon Y.; Ganapathi K.; Salahuddin S. How Good Can Monolayer MoS_2_ Transistors Be?. Nano Lett. 2011, 11 (9), 3768–3773. 10.1021/nl2018178. 21790188

[ref44] Henrotte O.; Bottein T.; Casademont H.; Jaouen K.; Bourgeteau T.; Campidelli S.; Derycke V.; Jousselme B.; Cornut R. Electronic Transport of MoS_2_ Monolayered Flakes Investigated by Scanning Electrochemical Microscopy. ChemPhysChem 2017, 18 (19), 2777–2781. 10.1002/cphc.201700343. 28771994

[ref45] Du H.-Y.; Huang Y.-F.; Wong D.; Tseng M.-F.; Lee Y.-H.; Wang C.-H.; Lin C.-L.; Hoffmann G.; Chen K.-H.; Chen L.-C. Nanoscale Redox Mapping at the MoS_2_-Liquid Interface. Nat. Commun. 2021, 12, 1321 10.1038/s41467-021-21660-z. 33637747 PMC7910562

[ref46] Yasaei P.; Kumar B.; Hantehzadeh R.; Kayyalha M.; Baskin A.; Repnin N.; Wang C.; Klie R. F.; Chen Y. P.; Král P.; Salehi-Khojin A. Chemical Sensing with Switchable Transport Channels in Graphene Grain Boundaries. Nat. Commun. 2014, 5, 4911 10.1038/ncomms5911. 25241799

[ref47] Veliev F.; Cresti A.; Kalita D.; Bourrier A.; Belloir T.; Briançon-Marjollet A.; Albrieux M.; Roche S.; Bouchiat V.; Delacour C. Sensing Ion Channel in Neuron Networks with Graphene Field Effect Transistors. 2D Mater. 2018, 5 (4), 045020 10.1088/2053-1583/aad78f.

[ref48] Kim S.; Park H.; Choo S.; Baek S.; Kwon Y.; Liu N.; Yang J. Y.; Yang C.-W.; Yoo G.; Kim S. Active-Matrix Monolithic Gas Sensor Array Based on MoS_2_ Thin-Film Transistors. Commun. Mater. 2020, 1, 86 10.1038/s43246-020-00086-y.

[ref49] Li M.; Biswas S.; Hail C. U.; Atwater H. A. Refractive Index Modulation in Monolayer Molybdenum Diselenide. Nano Lett. 2021, 21 (18), 7602–7608. 10.1021/acs.nanolett.1c02199. 34468150

[ref50] Back P.; Zeytinoglu S.; Ijaz A.; Kroner M.; Imamoǧlu A. Realization of an Electrically Tunable Narrow-Bandwidth Atomically Thin Mirror Using Monolayer MoSe_2_. Phys. Rev. Lett. 2018, 120 (3), 037401 10.1103/PhysRevLett.120.037401. 29400509

[ref51] Rigosi A. F.; Hill H. M.; Li Y.; Chernikov A.; Heinz T. F. Probing Interlayer Interactions in Transition Metal Dichalcogenide Heterostructures by Optical Spectroscopy: MoS_2_/WS_2_ and MoSe_2_/WSe_2_. Nano Lett. 2015, 15 (8), 5033–5038. 10.1021/acs.nanolett.5b01055. 26186085

