# Supplementary material for: In Operando Study of Charge Modulation in MoS2 Transistors by Excitonic Reflection Microscopy
Source: ACS Nano. 2024 Mar 28;18(14):9886–94. doi: 10.1021/acsnano.3c09337 (PMC11008581; doi:10.1021/acsnano.3c09337)
Supplement: Supplementary file 1 — nn3c09337_si_001.pdf [file nn3c09337_si_001.pdf]

Supporting Information for

In operando study of charge modulation in MoS_2 transistors by excitonic reflection microscopy

Nathan Ullberg*, Arianna Filoramo, Stéphane Campidelli, Vincent Derycke*

Université Paris-Saclay, CEA, CNRS, NIMBE, LICSEN, 91191, Gif-sur-Yvette, France

* To whom correspondence should be addressed:
nathan.ullberg@cea.fr, vincent.derycke@cea.fr

1. Alpha parameter

The alpha parameter as described by Zhu et al.[1] was fitted for both the 10^{-1} M H_2O/NaCl electrolyte and for the DEME-TFSI electrolyte. It was found that:

- $\alpha \approx 2.0 \times 10^4$ cm^{2}C^{-1} for 10^{-1} M H_2O/NaCl,
- $\alpha \approx 8.8 \times 10^4$ cm^{2}C^{-1} for DEME-TFSI.

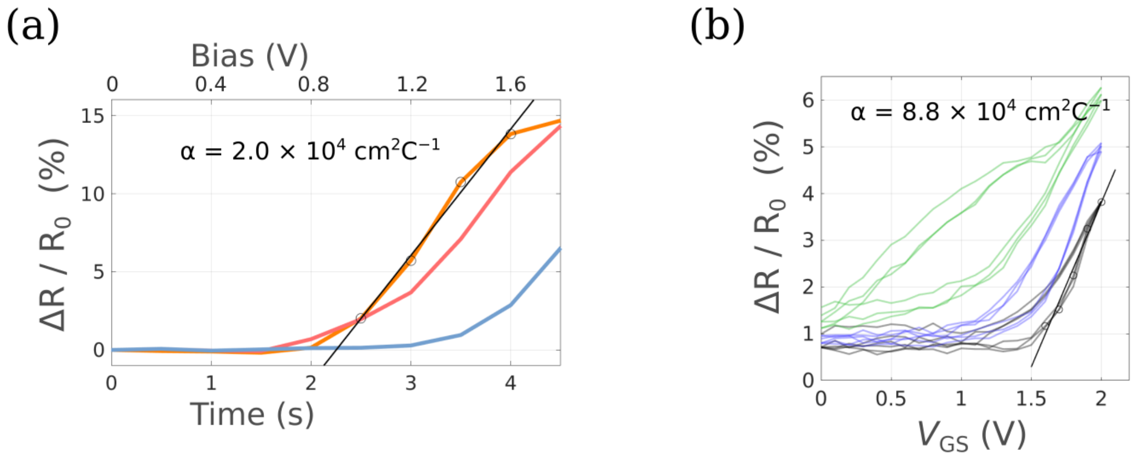

Figure S1: Linear fits of $\Delta R/R_0$ vs. gate bias, above threshold (V_{TH}), to extract the α-parameter used in the main text for the electrolytes (a) 10^{-1} M H_2O/NaCl and (b) DEME-TFSI.

2. Capacitance of DEME-TFSI

In Figures 3 and 4 of the main text, the ionic liquid (IL) DEME-TFSI was used. To estimate its electrical double layer capacitance (C_{EDL}), we fabricated MoS_2 FETs that could be controlled both through a solid-state back-gate with a well-defined capacitance C_{SiO2}, and by a top DEME-TFSI electrolytic gate (with a capacitance C_{EDL}). The source–drain electrode geometry is shown in Fig. S3(a). Using a back-gate oxide thickness of 150 nm and relative permittivity $\varepsilon_r(SiO_2)$ = 3.9, the back-gate capacitance per unit area is:

$$C_{\mathrm{SiO_2}} = \frac{\varepsilon_r \varepsilon_0}{d_{\mathrm{SiO_2}}} = \frac{3.9\varepsilon_0}{(150\ \mathrm{nm})} = 0.023\ \mu\mathrm{F/cm^2}$$

C_{EDL} can then be estimated by overlaying the transfer characteristic of both cases and multiplying V_{Si} by a factor that reveals C_{EDL}:

$$V_{\mathrm{IL}} = \frac{C_{\mathrm{SiO_2}}}{C_{\mathrm{EDL}}} V_{\mathrm{Si}} = \frac{0.023}{C_{\mathrm{EDL}}} V_{\mathrm{Si}}$$

We found that C_{EDL} = 0.8 μF/cm^2 provides a reasonable overlay. This value is similar, albeit lower, compared to the 1.55 μF/cm^2 value measured by Perera et al.[2].

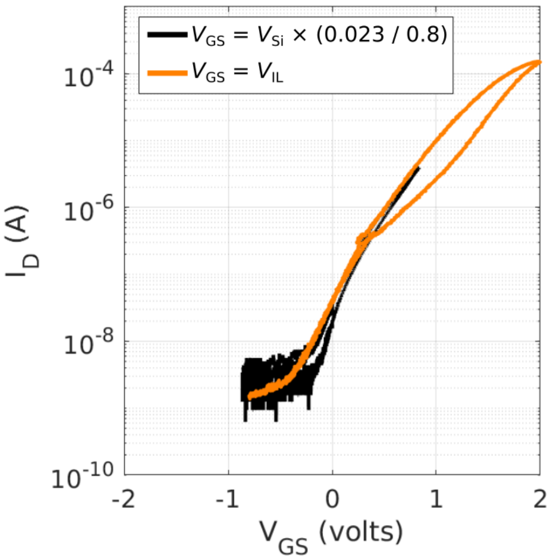

Figure S2: Overlay of the transfer characteristics of the same MoS_2 FETs controlled by the DEME-TFSI electrolytic gate (orange) and by the SiO_2/Si back-gate (black). The voltage range for the black curve was multiplied by a factor 0.023/0.8 which results in an estimate for C_{EDL}.

3. Electrical characteristic of DEME-TFSI gated MoS_2 FET

In order to obtain a representative electrical transfer characteristic of the DEME-TFSI (ionic liquid) gated 2D MoS_2 used in the experiments, an interdigitated FET configuration was realized on an a-SiO_2 / p^{++}-Si substrate, as shown by the scanning electron micrograph in Fig. S3(a). This interdigitated channel geometry helps in maximizing the MoS_2 channel current compared to the electrolyte conductivity. After application of the ionic liquid, the gate was swept at different drain values.

Fig. S3 (b) and (c) shows that for V_{DS} = 0.2 and 0.8 V, the gate is capable of an ON-OFF ratio of ~10^4. However for V_{DS} = 1.2 and 2.0 V it is clear that the drain is significantly participating in the switching of the device.

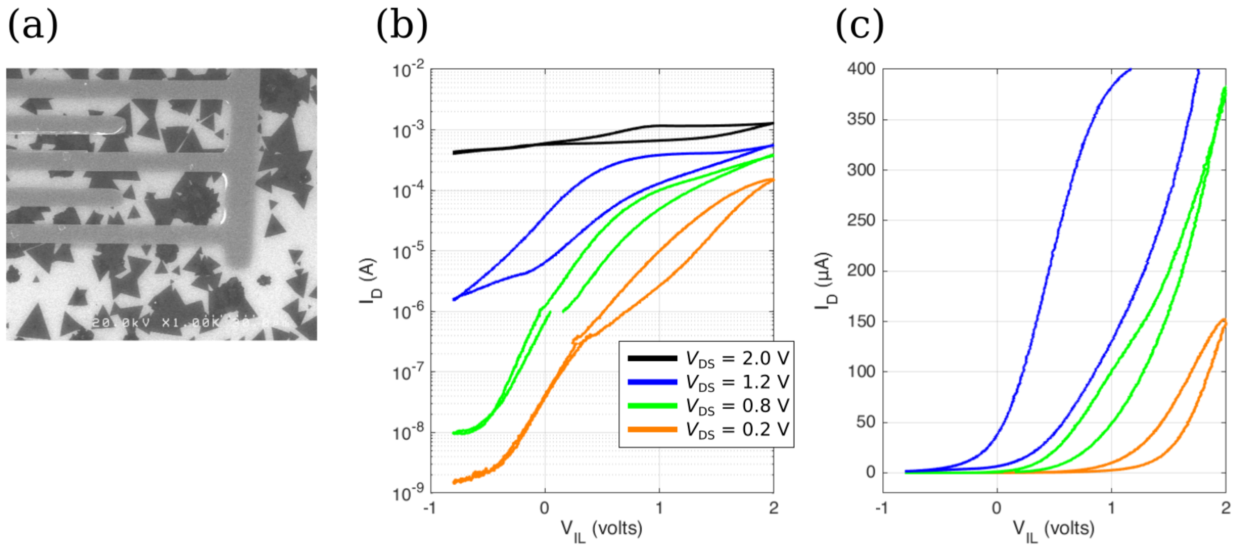

Figure S3: (a) Scanning electron micrograph of part of the channel of interdigitated MoS_2 FET on SiO_2/Si. (b) Transfer characteristic (in log scale) at different V_{DS} of the same FET operated by an electrolytic top-gate using DEME-TFSI as electrolyte. (c) Same as (b) but in linear scale.

4. Additional data for Figure 4

In the data for Fig. 4 of the main text, the cases V_{DS} = 0, 0.5, 1.5 V were done by first pulsing the drain, followed by oscillating linear gate sweeps at 200 mV/s in the range 0 to 2.0 V. The results as a function of time are shown in Fig. S4(b) for the center ROI in (a), with the $V_{DS}(t)$ and $V_{GS}(t)$ schematically in the upper pane and the $\Delta R/R_0(t)$ data in the lower pane. The corresponding false color images extracted from this data are indicated by the dashed lines to (d). Note that plotting of the $\Delta R/R_0$ curves as a function of V_{GS} instead of time is how Fig. 4(e) in the main text was attained.

The cases V_{DS} = 0.5, 1.0, 2.0 V were done by first pulsing the drain, followed by pulsing of the gate at 2.0 V each time. These pulses are represented schematically in the top three panes of Fig. S4(c). The middle panes show the $\Delta R/R_0$ vs. time response for each of the ROIs, including left, center and right. It is seen that the reflectance changes (and thus charge density changes) closest to the source are the most significant, which is expected and consistent with the result of Fig. 3 of the main text. Finally, dashed lines indicate the corresponding false color images for V_{DS}=1.0 and 2.0 V (orange and red). The blue line is dotted (instead of dashed like the others) because although the V_{DS}=0.5 V case was performed both in the linear-gate-sweep and pulsed-gate versions, the extracted false color image was done for the former case and not the latter.

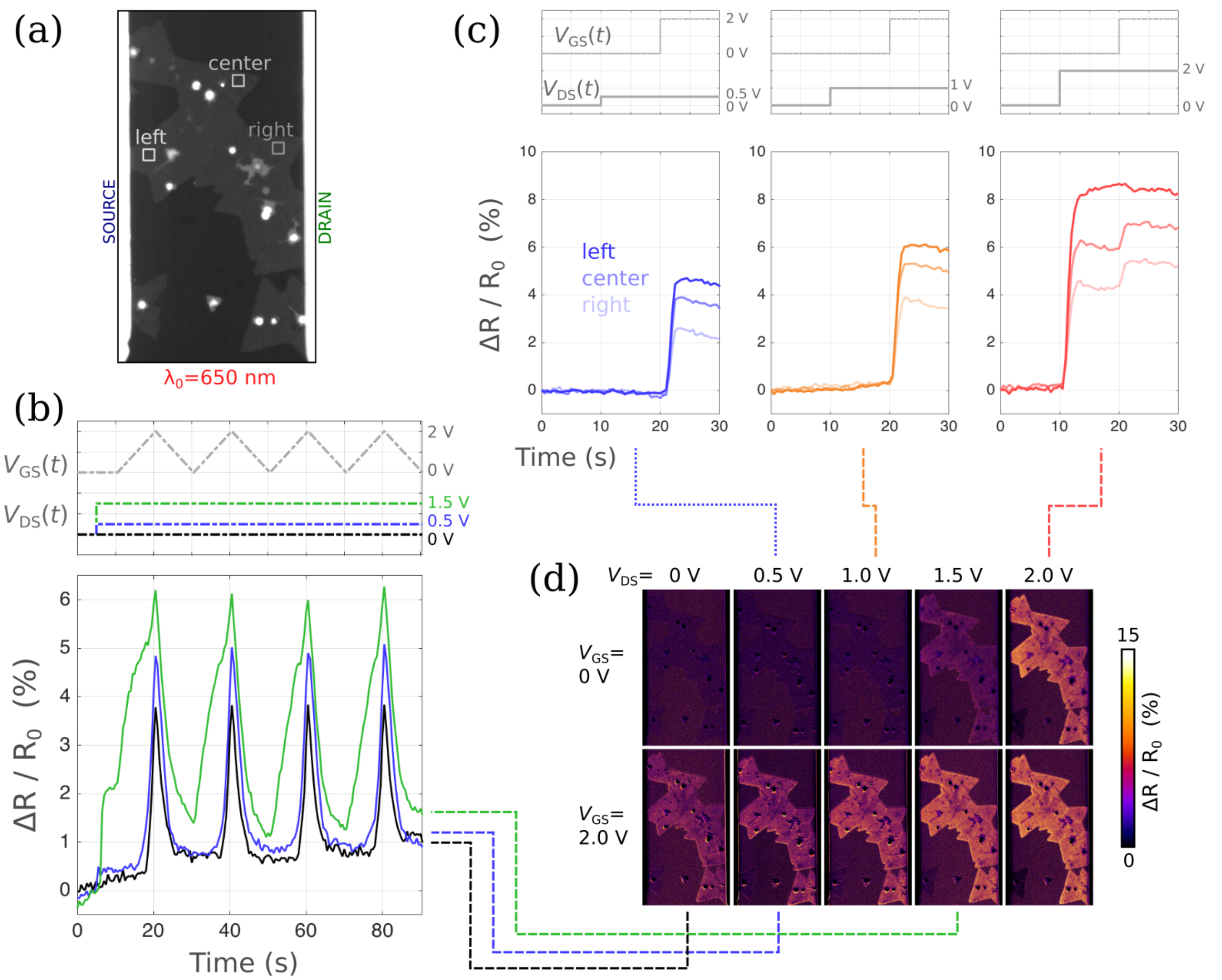

Figure S4: (a) ROIs of 1.47 × 1.47 μm^2 at left, center and right areas along the channel. (b) Top: schematic of the applied $V_{DS}(t)$ and $V_{GS}(t)$ for the cases V_{DS}=0, 0.5, 1.5 V. Bottom: $\Delta R/R_0$ versus time. (c) Top: Schematic of the applied $V_{DS}(t)$ and $V_{GS}(t)$ for the cases V_{DS}=0.5, 1.0, 2.0 V, with (middle) the corresponding 2-frames-per-second percent change reflectances and (bottom) the corresponding $\Delta R/R_0$ charge density images.

5. Effect of ROI size

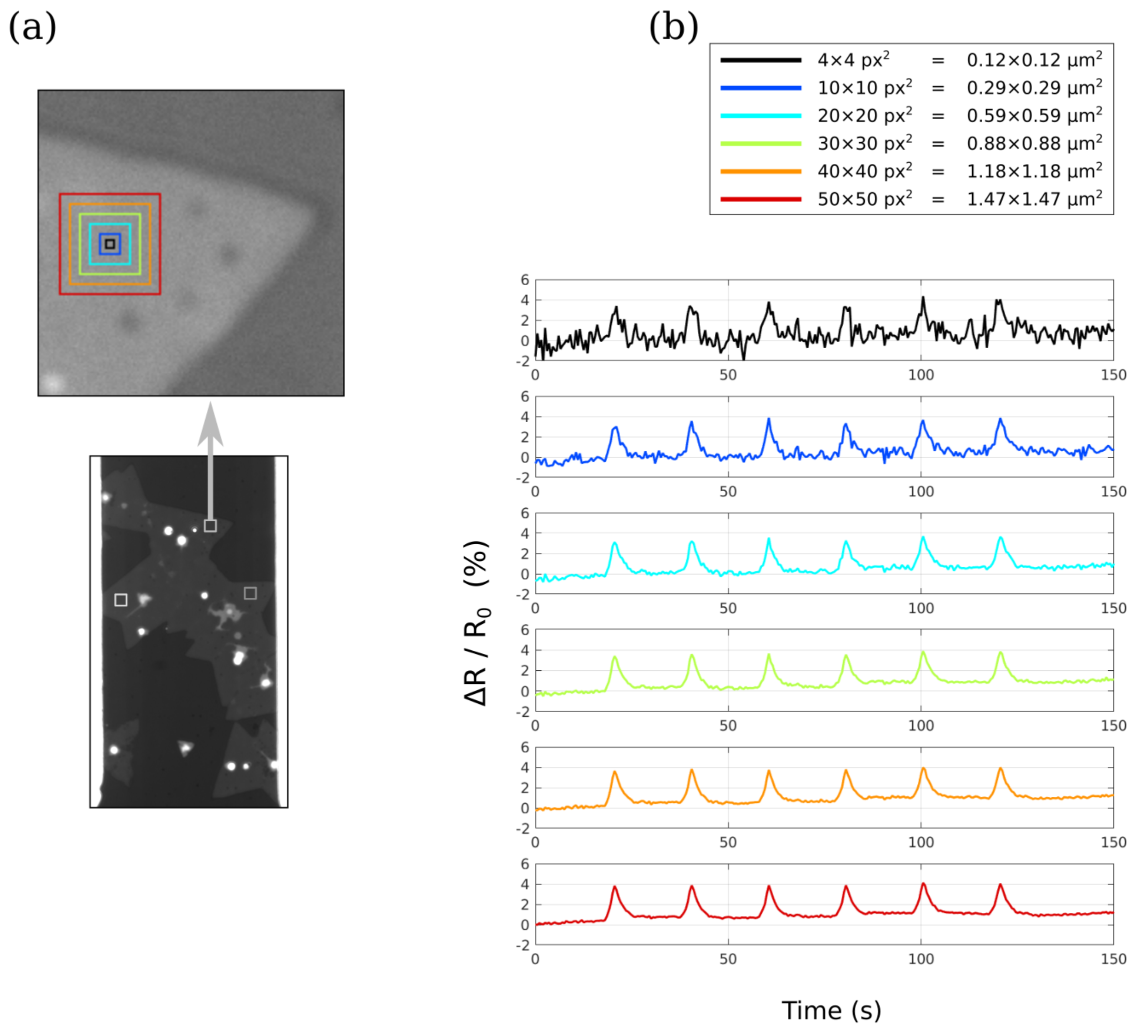

Figure S5: Effect of size of region of interest (ROI), tested for the cases indicated in (a) in the center of the channel (grayscale is 0–100 for top image). (b) V_{GS} was oscillated between 0 to 2 V at 200 mV/s, and resulting percent change reflectance curves are shown, with pixel and µm areas indicated in legend.

6. XRM at 12 bandpass filters

XRM was performed at 12 bandpass filters, including 400, 450, 550, 590, 600, 610, 620, 630, 640, 650, 660, 700 nm, each with 10 nm bandwidth, and an applied gate of 2 V. The resulting percent change reflectance images are shown in Fig. S6(a), but using always the minimum intensity as the reference, meaning that if $\Delta R/R_0 > 0$, then V_{GS}=0 was used as reference (as was done throughout the main text), whilst for $\Delta R/R_0 < 0$, V_{GS}=2 V was used as the reference. This was done to better visualize the magnitude of the changes.

The resultant $\Delta R/R_0$ XRM spectrum is plotted in (b) for all the ROIs as defined in (c), always using V_{GS}=0 V as reference in this case. As expected, only near excitonic wavelengths is there significant modulation in the reflectance. Interestingly though, some wavelengths such as 660 nm reveal more local variation, as evident both in (b) and in the associated image in (a). Although it is outside the scope of the work, it would be interesting to further research this aspect.

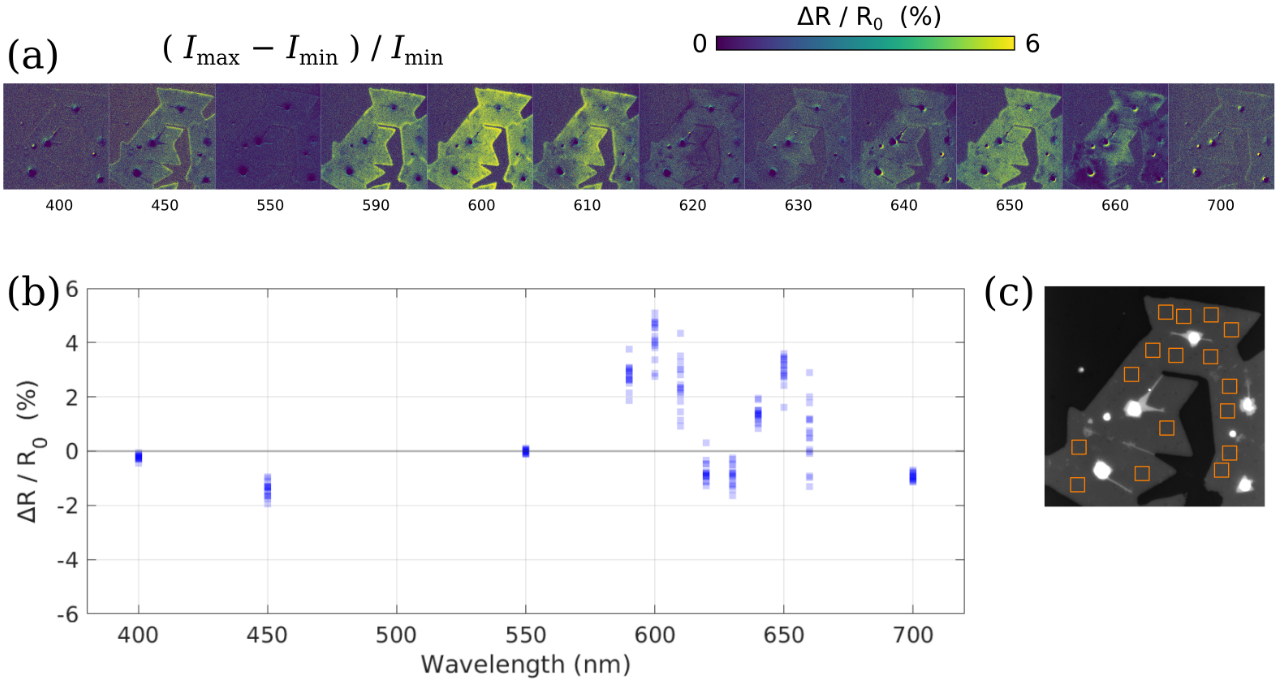

Figure S6: The same device as Fig. 3 was capacitively gated at 2 V, for 12 bandpass filters in the range 400–700 nm. (a) Percent change reflectance images using the minimum as reference for each case, then scaled from 0 to 6%. (b) Corresponding $\Delta R/R_0$ values vs. wavelength, for all ROIs indicated in (c).

7. Cleaning PMMA residues after transfer

The cleaning of samples after PMMA-based transfer was optimized to ensure that no residues would remain. In an early example, residues can be seen on the flakes, exemplified by the IRM color micrograph in Fig. S7(a) where the blue color is PMMA. By improving the recipe and adding multiple steps of acetone rinsing and acetone baths, transfers with no visible residue were realized, as exemplified in Fig. S7(b). The latter cleaning recipe was used for all samples and data presented in the article. (Note that in these particular examples, the MoS_2 is lying on an additional ~ 3 nm gold layer on the glass,[3] which does not change the conclusion.)

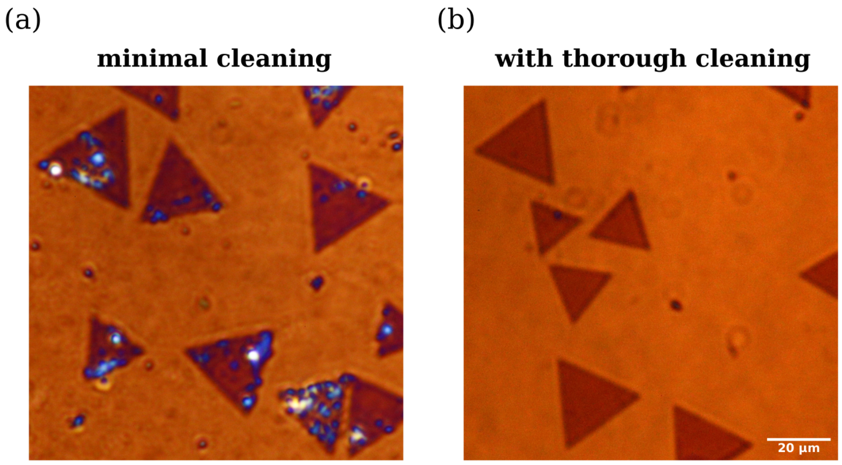

Figure S7: Two IRM color micrographs of MoS_2 monolayers with (a) minimal cleaning after transfer resulting in visible PMMA residues, and (b) thoroughly cleaned sample which successfully removed the PMMA.

8. Charging dynamics in grain boundaries

Two types of grain boundaries were discussed in the main text. In Fig. 2, we exemplified low-resistivity grain boundaries leading to homogeneous charging of connected flakes (Fig. 2(c) at 2.5s) in strong contrast with a high-resistivity grain boundary leading to a sizeable charging delay (Fig. 2(c) at 4.5s). We present below another example of a MoS_2 capacitor with grain boundaries. Compared to inter-flake boundaries as in Fig. 2, the grain boundaries seen in Fig. S8(a) (visible as bright protrusions) are boundaries within a single flake of complex morphology (i.e. crystalline domains originating from the same growth seed). A gate bias was cycled between 0 and +1V at a rate of 100 mV/s and the resulting percent change reflectivity for the ROIs defined in (a) are plotted in (b). The response of the grain boundary regions significantly differ from the monolayer response. While the monolayer charging is totally reversible, the reflectivity change at boundaries barely decay when the bias is ramped back from 1V to 0, which is a strong indication of charge trapping at such boundaries. Additionally, the trappings are visualized in (c) as a $\Delta R/R_0$ map, computed for the two frames indicated by dashed lines in (b). This experiment thus further highlight the strength of XRM for the study of trapping dynamics at grain boundaries.

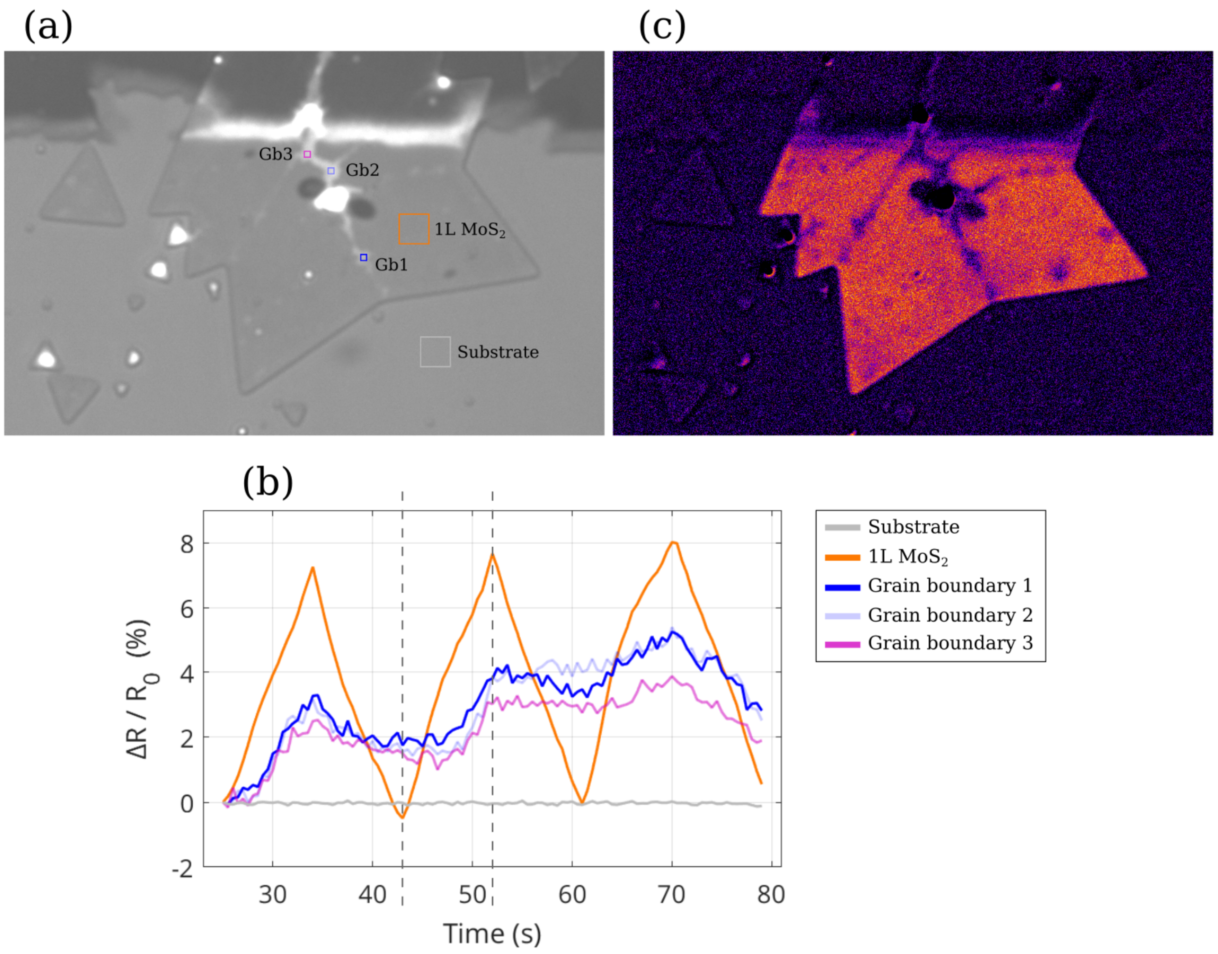

Figure S8: (a) IRM micrograph of a MoS_2 monolayer with multiple grain boundaries sitting on glass and connected by a Cr/Au layer on the top. ROIs have been defined at substrate, monolayer and 3 grain boundary regions. Grayscale was adjusted to (0,130) for better visibility. (b) Percent change reflectivity response for each ROI at λ = 650 nm, for three gating cycles between 0 and +1V at a rate of 100 mV/s using H_2O/NaCl as electrolyte. (c) XRM charge density image at +1V using 0V as reference at the video frames indicated by gray dashed lines in (b).

9. Hysteresis width evaluations from reflectivity plots

As stated in the main text, the optical $\Delta R/R_0(V_{GS})$ curves bear strong similarities with conventional electrical transfer characteristics $I_D(V_{GS})$. In particular, they both allow quantifying the hysteresis occurring during gate sweeps. To complement Fig. 4(e), where the gate was swept back and forth between 0 and 2 V for the drain potentials V_{DS} = 0, 0.5 and 1.5 volts, we reproduced in Fig. S9 below, the second cycle of these three sweeps and measured the hysteresis widths ΔV_{GS} in each case. For each width, the corresponding channel charge is calculated using the capacitance derived in Fig. S2 for this ionic liquid, namely C_{EDL} = 0.8 μF/cm^2. The result is ~ 1×10^{12}, 1.25×10^{12} and 3.25×10^{12} electrons/cm^2 respectively.

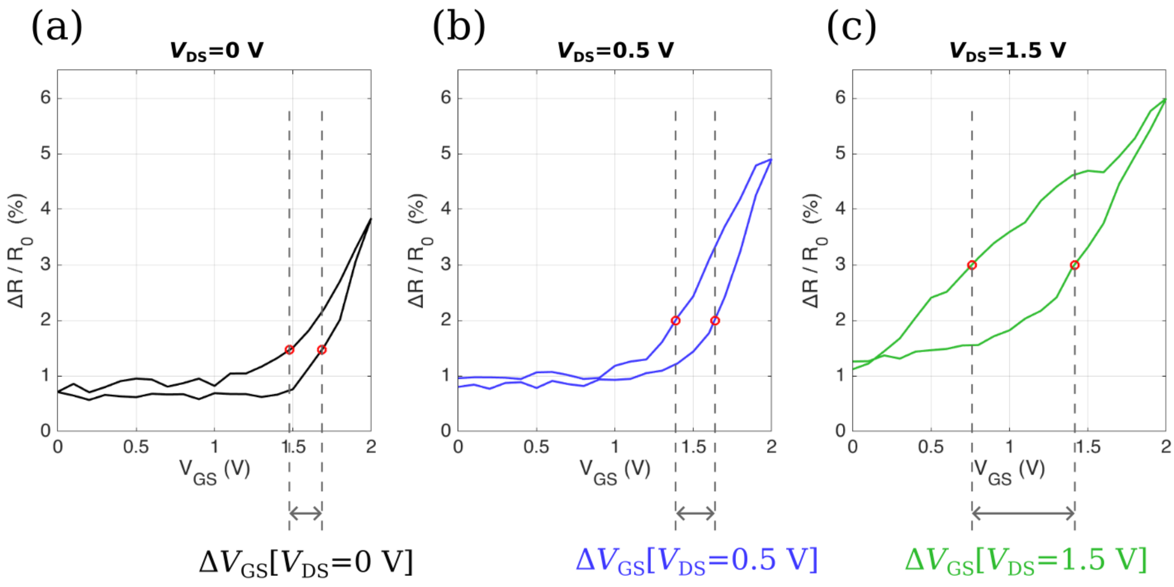

Figure S9: Second cycle of three gate voltage sweeps between 0 and 2 V, for the drain potentials (a) V_{DS}=0 V, (b) V_{DS}=0.5 V and (c) V_{DS}=1.5 V; with dashed lines and red markers indicating where hysteresis widths were extracted.

10. Hysteresis charge maps quantified

It is also possible to quantify and spatially resolve hysteresis effects due to charge trapping via XRM data. To illustrate this, the second cycle from the gate sweeps of Fig. 4(e) for the drain potentials of V_{DS}=0 V (black) and V_{DS}=1.5 V (green) have been reproduced below in Fig. S10(a). Examples of vertical cuts were chosen at V_{GS}=1.5 V for the former and V_{GS}=1 V for the latter, as indicated by the dashed lines and red markers. It is however important to note that such map can be produced at any polarization point (V_{DS}, V_{GS}) further illustrating the versatility of XRM. Using the relation derived earlier of $\Delta R/R_0 = \alpha\, \Delta Q$ and the value extracted for alpha of $\alpha = 8.8 \times 10^4$ cm^{2}C^{-1}, the charge for the two respective drain potentials for the center ROI indicated in Fig. 4(b) were found to be $\Delta Q(V_{DS}=0\text{ V}, V_{GS}=1.5\text{ V}) = 5.25 \times 10^{11}$ cm^{-2} and $\Delta Q(V_{DS}=1.5\text{ V}, V_{GS}=1\text{ V}) = 1.25 \times 10^{12}$ cm^{-2} . The complete charge maps for theses cases are shown in Fig. S10 (b) and (c). Interestingly, these maps are quite homogeneous, which was expected since the main sources of hysteresis (trap states in intrinsic MoS_2 defects such as sulfur vacancies, and trap stated at the glass/MoS_2 interface are distributed along in the whole FET channel).

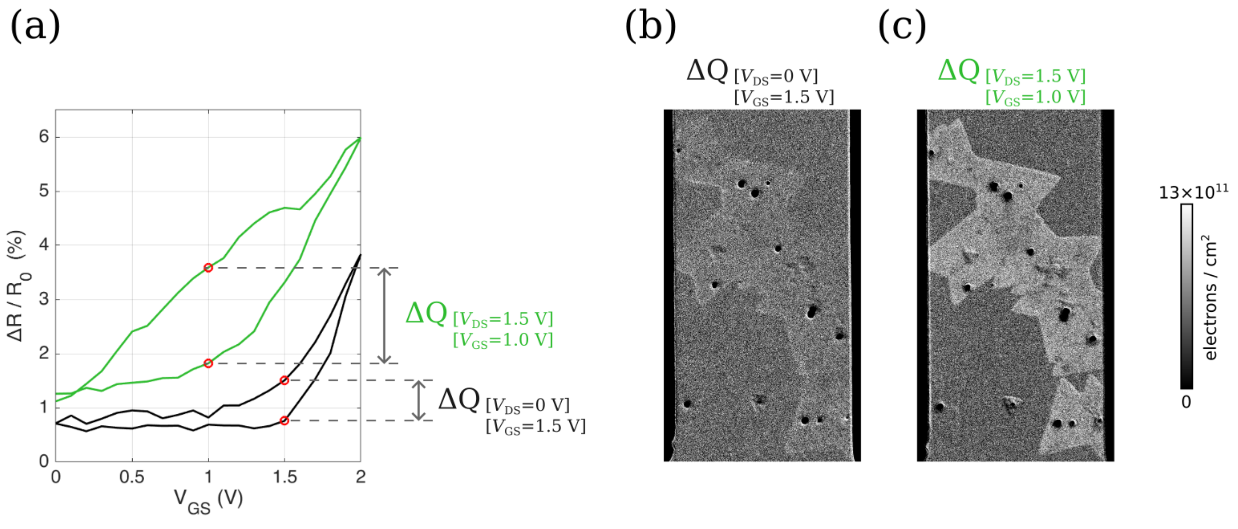

Figure S10: (a) Second cycle of three gate voltage sweeps between 0 and 2 V, for the drain potentials V_{DS}=0 V (black) and V_{DS}=1.5 V (green); with dashed lines and red markers indicating vertical cuts at V_{GS}=1.5 V and V_{GS}=1.0 V respectively. (b) and (c) show corresponding XRM charge maps at those vertical cuts.

11. Comparison of XRM with other techniques

In Table S11, XRM is compared with other techniques in terms of measured signal, lateral resolution and throughput. Corresponding references are included in the last column.

Technique	Measured signal	Lateral resolution	Throughput	Ref. applied to TMD devices
KPFM (kelvin probe force microscopy)	Work-function difference between tip and studied material	Based on tip sharpness (typically 30–50 nm)	Limited by scanning speed (typically a few minutes per image)	4, 5
C-AFM (conductive atomic force microscopy)	Current flow at the tip-sample contact point	Based on tip sharpness (typically 30–50 nm)	Limited by scanning speed (typically a few minutes per image)	6
SGM (scanning gate microscopy)	Sample electrical conductance as a function of tip position and tip potential	Based on tip sharpness (typically 30–50 nm)	Limited by scanning speed (typically a few minutes per image)	7
sMIM (scanning microwave impedance microscopy)	Local conductivity and local permittivity extracted from tip-sample admittance	Based on tip sharpness (typically 30–50 nm)	Limited by scanning speed (typically a few minutes per image)	8
μ-Raman (Raman spectrometry mapping)	Local doping and local stress extracted from Raman shifts	Laser spot size limited (~1μm)	Limited by Raman signal intensity and number of spectra per image (typically several hours per image)	9
μ-PL (photoluminescence mapping)	Photoluminescence intensity, spectral width and peak position	Laser spot size limited (~1μm)	Limited by PL signal intensity and number of spectra per image (typically one hour per image)	9
XRM (excitonic reflection microscopy)	Local charge density extracted from reflectivity change	Diffraction limited (~250 nm at the MoS_2 exciton energy and for NA=1.4)	0.5 second per image in this work, but throughput is only limited by the camera sensitivity	

Table S11: Characteristics of representative techniques able to map 2D transistors *in operando* (i.e. able to acquired local signal as a function of V_{DS} and V_{GS}). For the throughput criteria one image corresponds to one (V_{DS}, V_{GS}) polarization point.

1 Zhu, H.; Zhang, F.; Wang, H.; Lu, Z.; Chen, H.; Li, J.; Tao, N. Optical Imaging of Charges with Atomically Thin Molybdenum Disulfide. *ACS Nano* **2019**, *13* (2), 2298–2306. https://doi.org/10.1021/acsnano.8b09010.

2 Perera, M. M.; Lin, M.-W.; Chuang, H.-J.; Chamlagain, B. P.; Wang, C.; Tan, X.; Cheng, M. M.-C.; Tománek, D.; Zhou, Z. Improved Carrier Mobility in Few-Layer MoS_2 Field-Effect Transistors with Ionic-Liquid Gating. *ACS Nano* **2013**, *7* (5), 4449–4458. https://doi.org/10.1021/nn401053g.

3 Campidelli, S.; Abou Khachfe, R.; Jaouen, K.; Monteiller, J.; Amra, C.; Zerrad, M.; Cornut, R.; Derycke, V.; Ausserré, D. Backside Absorbing Layer Microscopy: Watching Graphene Chemistry. *Sci. Adv.* **2017**, *3* (5), e1601724. https://doi.org/10.1126/sciadv.1601724.

4 Vaknin, Y.; Dagan, R.; Rosenwaks, Y. Schottky Barrier Height and Image Force Lowering in Monolayer MoS_2 Field Effect Transistors. *Nanomaterials* **2020**, *10* (12), 2346. https://doi.org/10.3390/nano10122346.

5 Matković, A.; Petritz, A.; Schider, G.; Krammer, M.; Kratzer, M.; Karner-Petritz, E.; Fian, A.; Gold, H.; Gärtner, M.; Terfort, A.; Teichert, C.; Zojer, E.; Zojer, K.; Stadlober, B. Interfacial Band Engineering of MoS_2/Gold Interfaces Using Pyrimidine-Containing Self-Assembled Monolayers: Toward Contact-Resistance-Free Bottom-Contacts. *Adv. Electron. Mater.* **2020**, *6* (5), 2000110. https://doi.org/10.1002/aelm.202000110.

6 Giannazzo, F.; Bosi, M.; Fabbri, F.; Schilirò, E.; Greco, G.; Roccaforte, F. Direct Probing of Grain Boundary Resistance in Chemical Vapor Deposition-Grown Monolayer MoS_2 by Conductive Atomic Force Microscopy. *Phys. Status Solidi RRL* **2020**, *14* (2), 1900393. https://doi.org/10.1002/pssr.201900393.

7 Matsunaga, M.; Higuchi, A.; He, G.; Yamada, T.; Krüger, P.; Ochiai, Y.; Gong, Y.; Vajtai, R.; Ajayan, P. M.; Bird, J. P.; Aoki, N. Nanoscale-Barrier Formation Induced by Low-Dose Electron-Beam Exposure in Ultrathin MoS_2 Transistors. *ACS Nano* **2016**, *10* (10), 9730–9737. https://doi.org/10.1021/acsnano.6b05952.

8 Wu, D.; Li, X.; Luan, L.; Wu, X.; Li, W.; Yogeesh, M. N.; Ghosh, R.; Chu, Z.; Akinwande, D.; Niu, Q.; Lai, K. Uncovering Edge States and Electrical Inhomogeneity in MoS_2 Field-Effect Transistors. *Proc. Natl. Acad. Sci. U.S.A.* **2016**, *113* (31), 8583–8588. https://doi.org/10.1073/pnas.1605982113.

9 Michail, A.; Delikoukos, N.; Parthenios, J.; Galiotis, C.; Papagelis, K. Optical Detection of Strain and Doping Inhomogeneities in Single Layer MoS_2. *Appl. Phys. Lett.* **2016**, *108* (17), 173102. https://doi.org/10.1063/1.4948357.
